# Supplementary material for: Soil Salinization and Ancient Hulled Wheat: A Study on Antioxidant Defense Mechanisms
Source: Plants (Basel). 2025 Feb 22;14(5):678. doi: 10.3390/plants14050678 (PMC11901727; doi:10.3390/plants14050678)
Supplement: Supplementary file 1 [file plants-14-00678-s001.zip › Supplementary materials S2-statistics.pdf]

Soil Salinization and Ancient Hulled Wheat: A Study on Antioxidant Defense Mechanisms

Table S2.1. Tests of Between-Subjects Effects

Source	Dependent Variable	Type III Sum of Squares	df	Mean Square	F	P
Corrected Model	ProtCons	3433051.301[a]	151	22735.439	256.226	0.000
	SOD	8.006[b]	151	0.053	14.555	0.000
	CAT	.152[c]	151	0.001	2.119	0.000
	GR	1.812[d]	151	0.012	18.545	0.000
	GST	.635[e]	151	0.004	1.895	0.000
	APX	.839[f]	151	0.006	66.268	0.000
	Prolin	15673474.706[g]	151	103797.846	1027.182	0.000
	MDA	7312743.457[h]	151	48428.765	519.170	0.000
Wheats	ProtCons	104657.635	3	34885.878	393.160	0.000
	SOD	2.160	3	0.720	197.704	0.000
	CAT	0.032	3	0.011	22.260	0.000
	GR	0.749	3	0.250	385.800	0.000
	GST	0.108	3	0.036	16.169	0.000
	APX	0.141	3	0.047	561.399	0.000
	Prolin	299302.669	3	99767.556	987.298	0.000
	MDA	98286.581	3	32762.194	351.220	0.000
Sections	ProtCons	320259.935	1	320259.935	3609.290	0.000
	SOD	1.583	1	1.583	434.661	0.000
	CAT	0.019	1	0.019	40.024	0.000
	GR	0.163	1	0.163	252.276	0.000
	GST	0.015	1	0.015	6.761	0.010
	APX	0.016	1	0.016	191.173	0.000
	Prolin	122947.541	1	122947.541	1216.687	0.000
	MDA	3938.880	1	3938.880	42.226	0.000
Doses	ProtCons	2600832.407	18	144490.689	1628.392	0.000
	SOD	2.964	18	0.165	45.205	0.000
	CAT	0.029	18	0.002	3.348	0.000
	GR	0.627	18	0.035	53.795	0.000
	GST	0.206	18	0.011	5.159	0.000
	APX	0.516	18	0.029	341.949	0.000
	Prolin	14791327.211	18	821740.401	8131.928	0.000
	MDA	7067548.487	18	392641.583	4209.228	0.000
Wheats * Sections	ProtCons	564.264	3	188.088	2.120	0.098
	SOD	0.015	3	0.005	1.363	0.254
	CAT	0.006	3	0.002	4.482	0.004
	GR	0.060	3	0.020	30.993	0.000
	GST	0.011	3	0.004	1.584	0.193
	APX	0.002	3	0.001	8.426	0.000
	Prolin	3469.495	3	1156.498	11.445	0.000
	MDA	1511.190	3	503.730	5.400	0.001
Wheats * Doses	ProtCons	71916.512	54	1331.787	15.009	0.000
	SOD	0.580	54	0.011	2.949	0.000
	CAT	0.031	54	0.001	1.209	0.165
	GR	0.127	54	0.002	3.646	0.000
	GST	0.132	54	0.002	1.097	0.310
	APX	0.024	54	0.000	5.280	0.000
	Prolin	84834.147	54	1571.003	15.547	0.000
	MDA	57953.031	54	1073.204	11.505	0.000
Sections * Doses	ProtCons	322242.433	18	17902.357	201.757	0.000
	SOD	0.347	18	0.019	5.297	0.000
	CAT	0.009	18	0.000	1.035	0.420
	GR	0.039	18	0.002	3.342	0.000
	GST	0.038	18	0.002	0.939	0.532
	APX	0.124	18	0.007	82.036	0.000
	Prolin	304415.597	18	16911.978	167.361	0.000
	MDA	51304.605	18	2850.256	30.556	0.000
Wheats * Sections * Doses	ProtCons	19691.261	54	364.653	4.110	0.000
	SOD	0.348	54	0.006	1.768	0.002
	CAT	0.027	54	0.000	1.036	0.414
	GR	0.044	54	0.001	1.245	0.131
	GST	0.126	54	0.002	1.053	0.383
	APX	0.017	54	0.000	3.676	0.000
	Prolin	63122.571	54	1168.936	11.568	0.000
	MDA	21535.984	54	398.815	4.275	0.000

a. R Squared = 0.992 (Adjusted R Squared = 0.988); b. R Squared = 0.879 (Adjusted R Squared = 0.818); c. R Squared = 0.514 (Adjusted R Squared = 0.271); d. R Squared = 0.902 (Adjusted R Squared = 0.854); e. R Squared = 0.486 (Adjusted R Squared = 0.229); f. R Squared = 0.971 (Adjusted R Squared = 0.956); g. R Squared = 0.998 (Adjusted R Squared = 0.997); h. R Squared = 0.996 (Adjusted R Squared = 0.994); * df indicated degrees of freedom ** P, indicates significance

Table S2.2. Levene's Test of Equality of Error Variances[a]

		Levene Statistic	df1	df2	P
ProtCons	Based on Mean	3.498	151	303	0,000
	Based on Median	0.889	151	303	0,792
	Based on Median and with adjusted df	0.889	151	41,375	0,701
	Based on trimmed mean	3.227	151	303	0,000
SOD	Based on Mean	14.950	151	303	0,000
	Based on Median	0.996	151	303	0,504
	Based on Median and with adjusted df	0.996	151	3,894	0,594
	Based on trimmed mean	11.894	151	303	0,000
CAT	Based on Mean	15.586	151	303	0,000
	Based on Median	0.989	151	303	0,525
	Based on Median and with adjusted df	0.989	151	3,949	0,597
	Based on trimmed mean	12.303	151	303	0,000
GR	Based on Mean	15.233	151	303	0,000
	Based on Median	0.993	151	303	0,513
	Based on Median and with adjusted df	0.993	151	2,156	0,627
	Based on trimmed mean	12.081	151	303	0,000
GST	Based on Mean	15.751	151	303	0,000
	Based on Median	0.999	151	303	0,496
	Based on Median and with adjusted df	0.999	151	2,008	0,630
	Based on trimmed mean	12.432	151	303	0,000
APX	Based on Mean	10.793	151	303	0,000
	Based on Median	0.931	151	303	0,687
	Based on Median and with adjusted df	0.931	151	7,277	0,617
	Based on trimmed mean	8.947	151	303	0,000
Prolin	Based on Mean	11.183	151	303	0,000
	Based on Median	0.890	151	303	0,790
	Based on Median and with adjusted df	0.890	151	9,705	0,652
	Based on trimmed mean	9.168	151	303	0,000
MDA	Based on Mean	14.465	151	303	0,000
	Based on Median	0.972	151	303	0,573
	Based on Median and with adjusted df	0.972	151	2,089	0,637
	Based on trimmed mean	11.545	151	303	0,000
Tests the null hypothesis that the error variance of the dependent variable is equal across groups.					
a. Design: Intercept + Wheats + Sections + Doses + Wheats * Sections + Wheats * Doses + Sections * Doses + Wheats * Sections * Doses; * df indicated degrees of freedom ** P, indicates significance					

Table S2.3. MANOVA-Multivariate Tests[a]

Effect		Value	F	Hypothesis df	Error df	P
Intercept	Pillai's Trace	1.000	348447.595[b]	8.000	296.000	0.000
	Wilks' Lambda	0.000	348447.595[b]	8.000	296.000	0.000
	Hotelling's Trace	9417.503	348447.595[b]	8.000	296.000	0.000
	Roy's Largest Root	9417.503	348447.595[b]	8.000	296.000	0.000
Wheats	Pillai's Trace	1.888	63.267	24.000	894.000	0.000
	Wilks' Lambda	0.008	152.851	24.000	859.091	0.000
	Hotelling's Trace	27.265	334.757	24.000	884.000	0.000
	Roy's Largest Root	23.935	891.583[c]	8.000	298.000	0.000
Sections	Pillai's Trace	0.949	690.694[b]	8.000	296.000	0.000
	Wilks' Lambda	0.051	690.694[b]	8.000	296.000	0.000
	Hotelling's Trace	18.667	690.694[b]	8.000	296.000	0.000
	Roy's Largest Root	18.667	690.694[b]	8.000	296.000	0.000
Doses	Pillai's Trace	4.802	25.274	144.000	2424.000	0.000
	Wilks' Lambda	0.000	175.156	144.000	2191.384	0.000
	Hotelling's Trace	878.072	1794.254	144.000	2354.000	0.000
	Roy's Largest Root	604.403	10174.114[c]	18.000	303.000	0.000
Wheat * Sections	Pillai's Trace	0.469	6.907	24.000	894.000	0.000
	Wilks' Lambda	0.573	7.569	24.000	859.091	0.000
	Hotelling's Trace	0.671	8.243	24.000	884.000	0.000
	Roy's Largest Root	0.546	20.321[c]	8.000	298.000	0.000
Wheat * Doses	Pillai's Trace	3.549	4.475	432.000	2424.000	0.000
	Wilks' Lambda	0.004	5.568	432.000	2363.022	0.000
	Hotelling's Trace	10.190	6.941	432.000	2354.000	0.000
	Roy's Largest Root	3.697	20.745[c]	54.000	303.000	0.000
Sections * Doses	Pillai's Trace	3.660	14.198	144.000	2424.000	0.000
	Wilks' Lambda	0.000	30.374	144.000	2191.384	0.000
	Hotelling's Trace	29.806	60.905	144.000	2354.000	0.000
	Roy's Largest Root	15.781	265.644[c]	18.000	303.000	0.000
Wheats * Sections * Doses	Pillai's Trace	2.566	2.650	432.000	2424.000	0.000
	Wilks' Lambda	0.030	3.040	432.000	2363.022	0.000
	Hotelling's Trace	5.237	3.567	432.000	2354.000	0.000
	Roy's Largest Root	2.272	12.746[c]	54.000	303.000	0.000

a. Design: Intercept + Wheats + Sections + Doses + Wheats * Sections + Wheats * Doses + Sections * Doses + Wheats * Sections * Doses; * df indicated degrees of freedom ** P, indicates significance
