# Supplementary material for: Soil Salinization and Ancient Hulled Wheat: A Study on Antioxidant Defense Mechanisms
Source: Plants (Basel). 2025 Feb 22;14(5):678. doi: 10.3390/plants14050678 (PMC11901727; doi:10.3390/plants14050678)
Supplement: Supplementary file 1 [file plants-14-00678-s001.zip › Supplementary Materials- S1.pdf]

Soil Salinization and Ancient Hulled Wheat: A Study on Antioxidant Defense Mechanisms

Table S1.1. Effect of salt treatments on root development in hulled wheat

Individual salt treatments	*Wheats*	*Fresh weight (gr)*	*Dry weight (gr)*	*Dw/Fw (%)*
Control	*T. boeoticum*	3.40±0.62^{ab}	0.47±0.06^{c}	13.82
	T. dicoccum	5.20±0.10^{bc}	0.73±0.06^{d}	14.04
	T. monococcum	4.70±0.20^{b}	0.67±0.12^{d}	14.26
	T. speltoides	3.37±0.12^{ab}	0.43±0.06^{c}	12.76
30 mM NaCl	*T. boeoticum*	3.83±0.15^{b}	0.53±0.06^{c}	13.91
	T. dicoccum	5.13±0.06^{bc}	0.83±0.06^{e}	16.23
	T. monococcum	4.80±0.10^{b}	0.77±0.06^{d}	15.96
	T. speltoides	3.57±0.12^{ab}	0.47±0.06^{c}	13.06
50 mM NaCl	*T. boeoticum*	3.40±0.10^{ab}	0.50±0.00^{c}	14.71
	T. dicoccum	4.73±0.15^{b}	0.57±0.06^{c}	11.98
	T. monococcum	4.53±0.06^{b}	0.50±0.00^{c}	11.03
	T. speltoides	3.17±0.15^{a}	0.37±0.06^{b}	11.56
100 mM NaCl	*T. boeoticum*	3.17±0.06^{a}	0.20±0.00^{ab}	6.32
	T. dicoccum	4.43±0.15^{b}	0.37±0.06^{b}	8.28
	T. monococcum	4.03±0.06^{b}	0.30±0.00^{b}	7.44
	T. speltoides	2.90±0.10^{a}	0.17±0.06^{a}	5.72
150 mM NaCl	*T. boeoticum*	2.73±0.15^{a}	0.08±0.01^{a}	2.93
	T. dicoccum	4.10±0.10^{b}	0.19±0.01^{a}	4.63
	T. monococcum	3.83±0.06^{ab}	0.093±0.01^{a}	2.43
	T. speltoides	2.60±0.10^{a}	0.043±0.01^{a}	1.65
200 mM NaCl	*T. boeoticum*	2.53±0.06^{a}	0.07±0.02^{a}	2.76
	T. dicoccum	3.47±0.06^{ab}	0.19±0.02^{ab}	5.39
	T. monococcum	3.27±0.06^{a}	0.08±0.02^{a}	2.54
	T. speltoides	2.33±0.06^{a}	0.04±0.01^{a}	1.71
30 mM KCl	*T. boeoticum*	4.27±0.06^{b}	0.57±0.06^{c}	13.26
	T. dicoccum	5.07±0.06^{c}	0.77±0.06^{d}	15.14
	T. monococcum	4.73±0.06^{b}	0.57±0.06^{c}	11.98
	T. speltoides	3.57±0.06^{ab}	0.47±0.05^{c}	13.09
50 mM KCl	*T. boeoticum*	4.10±0.10^{b}	0.57±0.06^{c}	13.80
	T. dicoccum	4.47±0.02^{b}	0.63±0.05^{d}	14.17
	T. monococcum	4.20±0.01^{b}	0.43±0.05^{c}	10.31
	T. speltoides	3.53±0.06^{ab}	0.47±0.05^{c}	13.19
100 mM KCl	*T. boeoticum*	3.33±0.05^{ab}	0.43±0.05^{c}	12.99
	T. dicoccum	4.13±0.05^{b}	0.37±0.05^{b}	8.88
	T. monococcum	3.93±0.05^{b}	0.17±0.04^{a}	4.25
	T. speltoides	3.17±0.15^{a}	0.23±0.05^{ab}	7.36
150 mM KCl	*T. boeoticum*	2.97±0.05^{a}	0.14±0.02^{a}	4.72
	T. dicoccum	3.87±0.12^{b}	0.21±0.01^{ab}	5.43
	T. monococcum	3.50±0.10^{ab}	0.08±0.04^{a}	2.29
	T. speltoides	3.00±0.10^{a}	0.10±0.02^{a}	3.20
200 mM KCl	*T. boeoticum*	2.57±0.06^{a}	0.03±0.01^{a}	1.01
	T. dicoccum	3.33±0.06^{ab}	0.09±0.02^{a}	2.79
	T. monococcum	3.20±0.10^{a}	0.02±0.01^{a}	0.62
	T. speltoides	2.53±0.05^{a}	0.01±0.00^{a}	0.39

Combine Salt Applications	*Wheats*	*Fresh weight (gr)*	*Dry weight (gr)*	*Dw/Fw (%)*
50 mM NaCl + 50 mMKCl	*T. boeoticum*	3.40±0.10^{ab}	0.23±0.05^{b}	6.85
	T. dicoccum	4.90±0.10^{bc}	0.43±0.06^{c}	8.84
	T. monococcum	4.13±0.06^{b}	0.23±0.05^{c}	5.64
	T. speltoides	3.10±0.10^{a}	0.10±0.00^{a}	3.23
100 mM NaCl + 100 mM KCl	*T. boeoticum*	2.97±0.05^{a}	0.53±0.12^{c}	17.96
	T. dicoccum	4.63±0.05^{b}	0.70±0.10^{d}	15.11
	T. monococcum	3.97±0.12^{b}	0.53±0.06^{c}	13.44
	T. speltoides	2.80±0.00^{a}	0.40±0.01^{bc}	14.29
150 mM NaCl + 500 uM GB	*T. boeoticum*	2.90±0.10^{a}	0.14±0.02^{a}	4.69
	T. dicoccum	4.47±0.15^{b}	0.26±0.02^{b}	5.75
	T. monococcum	4.57±0.15^{b}	0.18±0.03^{a}	3.94
	T. speltoides	3.10±0.10^{a}	0.10±0.02^{a}	3.32
200 mM NaCl + 500 uM GB	*T. boeoticum*	2.93±0.15^{a}	0.11±0.02^{a}	3.85
	T. dicoccum	3.63±0.15^{ab}	0.23±0.02^{b}	6.41
	T. monococcum	3.73±0.15^{ab}	0.18±0.03^{a}	4.90
	T. speltoides	2.53±0.15^{a}	0.09±0.03^{a}	3.40
150 mM KCl + 500 uM GB	*T. boeoticum*	4.10±0.10^{b}	0.25±0.03^{b}	6.10
	T. dicoccum	4.80±0.20^{bc}	0.29±0.01^{b}	6.10
	T. monococcum	4.20±0.10^{b}	0.20±0.03^{ab}	4.67
	T. speltoides	3.63±0.15^{ab}	0.22±0.04^{b}	6.14
200 mM KCl + 500 uM GB	*T. boeoticum*	3.13±0.15^{a}	0.09±0.02^{a}	2.97
	T. dicoccum	4.30±0.20^{b}	0.15±0.03^{a}	3.49
	T. monococcum	3.90±0.10^{b}	0.06±0.02^{a}	1.54
	T. speltoides	3.10±0.10^{a}	0.05±0.02^{a}	1.62
50 mM NaCl + 50 mM KCl + 500 uM GB	*T. boeoticum*	4.20±0.20^{b}	0.87±0.03^{e}	20.79
	T. dicoccum	5.03±0.40^{c}	0.78±0.02^{de}	15.44
	T. monococcum	4.70±0.26^{b}	0.79±0.02^{de}	16.74
	T. speltoides	3.70±0.26^{ab}	0.63±0.03^{d}	17.11
100 mM NaCl + 100 mM KCl + 500 uM GB	*T. boeoticum*	4.33±0.21^{b}	0.34±0.02^{b}	7.92
	T. dicoccum	5.83±0.25^{c}	0.69±0.03^{d}	11.88
	T. monococcum	4.77±0.25^{b}	0.31±0.011^{b}	6.44
	T. speltoides	4.03±0.21^{b}	0.27±0.03^{b}	6.77

* Different letters in the columns indicate significant differences among wheat types ($p<0.05$).

Table S1.2. Effect of individual salt treatments on shoot development, chlorophyll and carotene in hulled wheat

Individual salt treatments	*Wheats*	*Fresh weight (gr)*	*Dry weight (gr)*	*Dw/Fw (%)*	*Plant height (cm)*	*Leaf lenght (cm)*	*Chl a (mg/gr fw)*	*Chl b (mg/gr fw)*	*Chl a/b*	*Total Chl (mg/gr fw)*	*Carotene (mg/gr fw)*
Control	*T. boeoticum*	26.93±0.85^{de}	3.40±0.10^{de}	12.62	23.67±0.33^{bc}	18.33±0.33^{ab}	2.04±0.01^{d}	0.90±0.01^{c}	2.27±0.01^{ef}	2.94±0.01^{d}	0.32±0.05^{bc}
	T. dicoccum	34.40±0.30^{h}	4.10±0.10^{gh}	11.92	30.33±0.33^{e}	21.67±0.33^{bc}	2.31±0.02^{e}	0.91±0.01^{c}	2.54±0.02^{fg}	3.23±0.02^{de}	0.21±0.01^{a}
	T. monococcum	30.87±0.61^{f}	3.83±0.06^{fg}	12.42	25.33±0.88^{c}	20.67±0.33^{b}	2.22±0.08^{e}	0.86±0.01^{bc}	2.58±0.05^{g}	3.09±0.08^{d}	0.28±0.01^{b}
	T. speltoides	24.90±0.44^{cd}	3.40±0.10^{de}	13.65	21.33±0.33^{b}	18.33±0.33^{ab}	2.14±0.01^{de}	0.90±0.01^{c}	2.38±0.01^{f}	3.04±0.02^{d}	0.33±0.05^{c}
30 mM NaCl	*T. boeoticum*	29.13±0.25^{ef}	4.10±0.10^{gh}	14.07	26.33±0.33^{cd}	19.67±0.33^{b}	2.59±0.02^{f}	0.99±0.01^{d}	2.62±0.02^{g}	3.58±0.01^{ef}	0.38±0.05^{cd}
	T. dicoccum	36.63±0.42^{i}	4.80±0.10^{k}	13.10	37.00±0.57^{g}	23.67±0.33^{c}	3.08±0.01^{gh}	0.97±0.01^{cd}	3.18±0.01^{hi}	4.06±0.01^{fg}	0.31±0.01^{bc}
	T. monococcum	35.80±0.20^{hi}	4.47±0.06^{ij}	12.48	31.67±0.33^{e}	20.33±0.33^{b}	2.78±0.01^{fg}	0.92±0.01^{c}	3.02±0.01^{h}	3.69±0.01^{ef}	0.33±0.01^{c}
	T. speltoides	26.87±0.42^{de}	3.90±0.10^{fg}	14.52	24.00±0.57^{c}	18.33±0.33^{ab}	2.64±0.02^{f}	0.99±0.01^{d}	2.67±0.02^{g}	3.63±0.03^{ef}	0.40±0.05^{d}
50 mM NaCl	*T. boeoticum*	29.77±0.38^{ef}	4.27±0.06^{hi}	14.33	27.33±0.33^{d}	18.67±0.33^{ab}	2.64±0.01^{f}	1.09±0.01^{e}	2.42±0.01^{f}	3.73±0.02^{f}	0.57±0.03^{g}
	T. dicoccum	36.60±0.10^{i}	5.03±0.05^{lm}	13.75	38.67±0.66^{gh}	24.67±0.33^{cd}	3.34±0.02^{h}	1.00±0.01^{d}	3.34±0.02^{i}	4.35±0.02^{gh}	0.33±0.01^{c}
	T. monococcum	38.37±0.15^{j}	4.77±0.05^{k}	12.42	32.33±0.33^{ef}	21.00±0.57^{b}	2.97±0.01^{g}	0.95±0.01^{cd}	3.13±0.01^{hi}	3.92±0.02^{fg}	0.40±0.01^{d}
	T. speltoides	27.87±0.25^{de}	4.00±0.10^{g}	14.35	25.33±0.33^{c}	17.33±0.33^{a}	2.87±0.01^{g}	1.09±0.02^{e}	2.63±0.02^{g}	3.96±0.016^{fg}	0.55±0.08^{fg}
100 mM NaCl	*T. boeoticum*	26.37±0.55^{d}	3.97±0.05^{g}	15.05	25.67±0.33^{c}	17.33±0.33^{a}	1.95±0.01^{d}	1.01±0.01^{d}	1.93±0.01^{d}	2.97±0.01^{d}	0.60±0.03^{gh}
	T. dicoccum	35.27±0.31h^{i}	4.80±0.10^{k}	13.61	36.33±0.33^{g}	21.67±0.33^{bc}	2.05±0.02^{d}	0.94±0.01^{c}	2.18±0.02^{e}	2.98±0.02^{d}	0.31±0.01^{bc}
	T. monococcum	37.37±0.06^{ij}	4.57±0.05^{ij}	12.22	30.67±0.33^{e}	19.67±0.33^{b}	2.19±0.01^{de}	0.95±0.01^{cd}	2.31±0.01^{ef}	3.14±0.01^{de}	0.37±0.01^{cd}
	T. speltoides	26.10±0.30^{cd}	3.80±0.10^{f}	14.56	24.33±0.33^{c}	16.33±0.33^{a}	1.81±0.01^{cd}	1.00±0.02^{d}	1.81±0.02^{cd}	2.82±0.02^{cd}	0.58±0.05^{g}
150 mM NaCl	*T. boeoticum*	25.57±0.21^{cd}	3.50±0.10^{e}	13.69	24.33±0.33^{c}	21.67±0.33^{bc}	1.53±0.01^{bc}	0.97±0.01^{cd}	1.58±0.01^{c}	2.51±0.01^{c}	0.56±0.05^{fg}
	T. dicoccum	34.03±0.21^{gh}	4.50±0.10^{j}	13.22	35.33±0.33^{fg}	23.67±0.33^{c}	1.28±0.02^{ab}	0.91±0.01^{c}	1.41±0.02^{b}	2.19±0.01^{b}	0.32±0.01^{bc}
	T. monococcum	36.57±0.06^{i}	4.43±0.05^{ij}	12.12	29.67±0.33^{de}	18.67±0.33^{ab}	1.15±0.01^{a}	0.93±0.01^{c}	1.24±0.01^{ab}	2.39±0.01^{bc}	0.37±0.01^{cd}
	T. speltoides	24.90±0.44^{cd}	3.17±0.15^{c}	12.71	20.67±0.33^{ab}	19.33±0.33^{b}	1.58±0.02^{bc}	0.96±0.02^{cd}	1.65±0.02^{c}	2.54±0.03^{c}	0.55±0.04^{fg}
200 mM NaCl	*T. boeoticum*	20.53±0.40^{a}	3.23±0.06^{cd}	15.75	23.33±0.33^{bc}	19.33±0.33^{b}	1.38±0.01^{b}	0.93±0.01^{c}	1.48±0.01^{b}	2.31±0.01^{bc}	0.56±0.03^{fg}
	T. dicoccum	28.76±0.35^{e}	4.03±0.05^{gh}	14.02	26.67±0.33^{cd}	22.67±0.33^{c}	0.89±0.02^{a}	0.86±0.01^{bc}	1.03±0.02^{a}	1.74±0.01^{a}	0.28±0.01^{b}
	T. monococcum	30.83±0.25^{fg}	3.60±0.10^{e}	11.68	25.67±0.33^{c}	17.67±0.33^{a}	1.19±0.02^{a}	0.89±0.01^{c}	1.34±0.02^{ab}	2.08±0.02^{ab}	0.35±0.01^{c}
	T. speltoides	19.83±0.35^{a}	3.03±0.06^{bc}	15.29	19.33±0.33^{a}	19.33±0.33^{b}	1.39±0.02^{b}	0.92±0.03^{c}	1.51±0.03^{b}	2.31±0.03^{bc}	0.56±0.05^{fg}
30 mM KCl	*T. boeoticum*	29.63±0.15^{ef}	3.23±0.64^{cd}	10.91	24.33±0.33^{c}	20.67±0.33^{b}	2.25±0.01^{e}	0.94±0.01^{c}	2.39±0.01^{f}	3.19±0.01^{de}	0.39±0.06^{d}
	T. dicoccum	36.57±0.20^{i}	4.53±0.06^{ij}	12.40	32.67±0.33^{ef}	24.33±0.33^{cd}	2.84±0.01^{g}	0.92±0.01^{c}	3.09±0.01^{hi}	3.76±0.01^{f}	0.31±0.01^{bc}
	T. monococcum	34.80±0.20^{h}	4.23±0.05^{hi}	12.16	30.67±0.33^{e}	21.00±0.57^{b}	2.60±0.02^{f}	0.88±0.01^{bc}	2.95±0.02^{h}	3.48±0.02^{e}	0.33±0.01^{c}
	T. speltoides	28.20±0.20^{e}	3.33±0.05^{d}	11.82	21.33±0.33^{b}	19.67±0.33^{b}	2.34±0.01^{e}	0.93±0.01^{c}	2.52±0.01^{fg}	3.28±0.02^{de}	0.38±0.05^{cd}
50 mM KCl	*T. boeoticum*	29.67±0.38^{ef}	3.63±0.05^{e}	12.25	23.33±0.33^{bc}	19.33±0.33^{b}	2.79±0.01^{fg}	1.00±0.03^{d}	2.79±0.02^{gh}	3.80±0.01^{f}	0.55±0.03^{fg}
	T. dicoccum	38.83±0.21^{j}	5.07±0.06^{lm}	13.05	33.67±0.33^{f}	23.67±0.33^{c}	3.14±0.01^{h}	0.94±0.01^{c}	3.34±0.01^{i}	4.08±0.01^{fg}	0.24±0.10^{ab}
	T. monococcum	36.56±0.57^{i}	4.83±0.06^{k}	13.22	32.33±0.33^{ef}	22.33±0.33^{c}	2.78±0.02^{fg}	0.90±0.01^{c}	3.09±0.02^{hi}	3.69±0.02^{ef}	0.38±0.01^{cd}
	T. speltoides	28.33±0.20^{e}	3.27±0.05^{cd}	11.53	21.33±0.33^{b}	19.33±0.33^{b}	2.86±0.01^{g}	0.98±0.01^{cd}	2.92±0.01^{h}	3.85±0.02^{f}	0.51±0.05^{f}
100 mM KCl	*T. boeoticum*	27.47±0.35^{de}	3.90±0.10^{g}	14.20	20.67±0.33^{ab}	18.67±0.33^{ab}	2.39±0.01^{e}	1.02±0.01^{d}	2.34±0.01^{ef}	3.41±0.01^{e}	0.67±0.08^{h}
	T. dicoccum	37.23±0.32^{ij}	5.17±0.06^{m}	13.88	32.33±0.33^{ef}	22.67±0.33^{c}	2.19±0.02^{de}	0.89±0.01^{c}	2.46±0.02^{f}	3.09±0.02^{d}	0.38±0.01^{cd}
	T. monococcum	36.23±0.21^{i}	4.67±0.15^{j}	12.88	30.33±0.33^{e}	21.33±0.33^{bc}	2.24±0.02^{e}	0.91±0.01^{c}	2.46±0.02^{f}	3.15±0.02^{de}	0.41±0.01^{d}
	T. speltoides	26.36±0.51^{d}	3.57±0.12^{e}	13.52	20.33±0.33^{ab}	17.67±0.33^{a}	2.19±0.02^{d}	1.01±0.02^{d}	2.17±0.02^{e}	3.20±0.03^{de}	0.62±0.03^{gh}
150 mM KCl	*T. boeoticum*	25.23±0.42^{cd}	3.20±0.10^{c}	12.68	19.67±0.33^{a}	16.67±0.33^{a}	2.25±0.01^{e}	0.87±0.01^{bc}	2.59±0.01^{g}	3.12±0.01^{de}	0.65±0.05^{h}
	T. dicoccum	36.50±0.30^{i}	4.57±0.05^{ij}	12.51	29.33±0.33^{de}	19.33±0.33^{b}	1.58±0.01^{bc}	0.81±0.01^{b}	1.95±0.01^{d}	2.39±0.01^{bc}	0.36±0.01^{cd}
	T. monococcum	35.43±0.21^{hi}	4.40±0.10^{i}	12.42	27.67±0.66^{d}	17.67±0.33^{a}	1.84±0.01^{cd}	0.83±0.01^{b}	2.22±0.01^{e}	2.68±0.01^{c}	0.40±0.01^{d}
	T. speltoides	24.30±0.17^{c}	2.87±0.12^{ab}	11.79	19.67±0.33^{a}	16.00±0.00^{a}	2.13±0.01^{de}	0.85±0.01^{bc}	2.47±0.01^{f}	2.99±0.02^{d}	0.61±0.08^{gh}
200 mM KCl	*T. boeoticum*	21.50±0.66^{ab}	2.93±0.05^{ab}	13.64	17.67±0.33^{a}	16.33±0.33^{a}	2.07±0.02^{d}	0.80±0.02^{b}	2.59±0.02^{g}	2.88±0.02^{cd}	0.63±0.08^{h}
	T. dicoccum	31.00±0.10^{fg}	4.20±0.10^{h}	13.55	26.67±0.33^{cd}	17.33±0.33^{a}	1.42±0.01^{b}	0.69±0.01^{a}	2.06±0.01^{de}	2.11±0.01^{b}	0.36±0.01^{cd}
	T. monococcum	30.63±0.15^{f}	4.00±0.10^{g}	13.06	25.00±0.57^{c}	16.33±0.33^{a}	1.58±0.01b^{c}	0.71±0.01^{a}	2.23±0.01^{e}	2.29±0.01^{bc}	0.40±0.01^{d}
	T. speltoides	22.23±0.51^{b}	2.60±0.10^{a}	11.69	18.33±0.33^{a}	15.33±0.33^{a}	1.97±0.01^{d}	0.78±0.02^{ab}	2.53±0.02^{fg}	2.75±0.03^{cd}	0.59±0.08^{g}

* Different letters in the columns indicate significant differences among wheat types ($p<0.05$).

Table S1.3. Effect of combined salt treatments on shoot development, chlorophyll and carotene in hulled wheat

Combine salt treatments	***Wheats***	***Fresh weight (gr)***	***Dry weight (gr)***	***Dw/Fw (%)***	***Plant height (cm)***	***Leaf lenght (cm)***	***Chl a (mg/gr fw)***	***Chl b (mg/gr fw)***	***Chl a/b***	***Total Chl (mg/gr fw)***	***Carotene (mg/gr fw)***
50 mM NaCl + 50 mM KCl	*T. boeoticum*	28.27 ± 0.57^{bc}	3.77 ± 0.05^{ab}	13.32	23.67 ± 0.33^{ab}	21.33 ± 0.33^{b}	2.75 ± 0.02^{f}	0.70 ± 0.33^{a}	3.93 ± 0.03^{h}	3.45 ± 0.35^{f}	0.73 ± 0.02^{de}
	T. dicoccum	39.13 ± 0.45^{e}	5.13 ± 0.05^{bc}	13.12	35.33 ± 0.33^{c}	24.67 ± 0.33^{c}	3.12 ± 0.02^{g}	0.92 ± 0.01^{bc}	3.39 ± 0.02^{f}	4.04 ± 0.02^{gh}	0.41 ± 0.01^{b}
	T. monococcum	38.00 ± 0.20^{de}	5.07 ± 0.06^{bc}	13.33	32.67 ± 0.33^{bc}	22.00 ± 0.57^{b}	2.91 ± 0.03^{fg}	0.92 ± 0.01^{bc}	3.16 ± 0.02^{ef}	3.83 ± 0.03^{g}	0.44 ± 0.01^{b}
	T. speltoides	27.40 ± 0.26^{bc}	3.53 ± 0.05^{a}	12.89	22.00 ± 0.57^{ab}	19.33 ± 0.33^{ab}	2.68 ± 0.02^{f}	1.01 ± 0.02^{de}	2.65 ± 0.02^{d}	3.69 ± 0.03^{fg}	0.63 ± 0.02^{d}
100 mM NaCl + 100 mM KCl	*T. boeoticum*	25.23 ± 0.32^{b}	3.36 ± 0.15^{a}	13.34	18.00 ± 0.01^{a}	17.33 ± 0.33^{a}	2.48 ± 0.02^{e}	1.05 ± 0.03^{e}	2.36 ± 0.03^{c}	3.53 ± 0.02^{f}	0.73 ± 0.02^{de}
	T. dicoccum	37.37 ± 0.21^{de}	4.50 ± 0.10^{b}	12.04	28.33 ± 0.33^{b}	21.67 ± 0.33^{b}	2.13 ± 0.01^{de}	0.91 ± 0.01^{bc}	2.34 ± 0.01^{c}	3.04 ± 0.01^{d}	0.44 ± 0.01^{b}
	T. monococcum	35.83 ± 0.15^{d}	4.20 ± 0.10^{b}	11.72	29.33 ± 0.33^{b}	20.33 ± 0.33^{ab}	2.34 ± 0.01^{e}	0.93 ± 0.01^{c}	2.52 ± 0.01^{d}	3.28 ± 0.01^{e}	0.50 ± 0.01^{bc}
	T. speltoides	24.63 ± 0.21^{b}	3.17 ± 0.15^{a}	12.85	18.33 ± 0.33^{a}	17.33 ± 0.33^{a}	2.34 ± 0.01^{e}	0.70 ± 0.04^{a}	3.34 ± 0.02^{f}	3.04 ± 0.06^{d}	0.70 ± 0.02^{d}
150 mM NaCl + 500 uM GB	*T. boeoticum*	25.66 ± 0.55^{b}	4.47 ± 0.06^{b}	17.40	28.00 ± 0.33^{b}	24.67 ± 0.33^{c}	1.61 ± 0.01^{c}	1.03 ± 0.02^{e}	1.56 ± 0.02^{b}	2.64 ± 0.01^{c}	0.72 ± 0.02^{de}
	T. dicoccum	34.46 ± 0.40^{d}	5.97 ± 0.15^{d}	17.31	35.33 ± 0.33^{c}	27.00 ± 0.57^{d}	1.39 ± 0.01^{b}	0.93 ± 0.01^{c}	1.49 ± 0.01^{b}	2.33 ± 0.01^{b}	0.35 ± 0.01^{a}
	T. monococcum	37.00 ± 0.46^{de}	5.30 ± 0.10^{c}	14.32	35.00 ± 0.57^{c}	21.67 ± 0.33^{b}	1.52 ± 0.01^{bc}	0.94 ± 0.01^{c}	1.62 ± 0.01^{b}	2.47 ± 0.01^{b}	0.42 ± 0.01^{b}
	T. speltoides	25.73 ± 0.21^{b}	3.76 ± 0.15^{ab}	14.63	24.00 ± 0.57^{ab}	23.33 ± 0.33^{bc}	1.43 ± 0.04^{b}	0.99 ± 0.01^{d}	1.44 ± 0.02^{b}	2.42 ± 0.04^{b}	0.69 ± 0.02^{d}
200 mM NaCl + 500 uM GB	*T. boeoticum*	21.30 ± 0.40^{a}	4.10 ± 0.30^{b}	19.25	28.67 ± 0.33^{b}	22.00 ± 0.58^{b}	1.56 ± 0.01^{bc}	0.98 ± 0.01^{d}	1.59 ± 0.01^{b}	2.55 ± 0.03^{bc}	0.77 ± 0.02^{e}
	T. dicoccum	29.47 ± 0.35^{bc}	4.87 ± 0.21^{bc}	16.52	31.67 ± 0.33^{bc}	25.33 ± 0.33^{c}	1.07 ± 0.01^{a}	0.90 ± 0.01^{bc}	1.19 ± 0.01^{a}	1.97 ± 0.01^{a}	0.35 ± 0.01^{a}
	T. monococcum	31.63 ± 0.59^{c}	5.00 ± 0.20^{bc}	15.81	30.33 ± 0.33^{bc}	22.00 ± 0.57^{b}	1.36 ± 0.01^{b}	0.92 ± 0.01^{bc}	1.48 ± 0.01^{b}	2.28 ± 0.01^{b}	0.43 ± 0.01^{b}
	T. speltoides	20.70 ± 0.46^{a}	3.83 ± 0.32^{ab}	18.52	24.33 ± 0.33^{ab}	21.33 ± 0.33^{b}	1.52 ± 0.01^{bc}	0.99 ± 0.01^{d}	1.54 ± 0.01^{b}	2.51 ± 0.02^{b}	0.74 ± 0.02^{de}
150 mM KCl + 500 uM GB	*T. boeoticum*	25.46 ± 0.70^{b}	4.10 ± 0.30^{b}	16.10	23.33 ± 0.33^{ab}	22.33 ± 0.33^{bc}	2.42 ± 0.01^{e}	0.91 ± 0.01^{bc}	2.66 ± 0.01^{d}	3.33 ± 0.01^{e}	0.86 ± 0.03^{ef}
	T. dicoccum	36.77 ± 0.65^{d}	5.56 ± 0.32^{c}	15.14	34.33 ± 0.33^{c}	23.67 ± 0.33^{c}	1.95 ± 0.01^{d}	0.86 ± 0.01^{b}	2.27 ± 0.01^{c}	2.81 ± 0.01^{cd}	0.44 ± 0.01^{b}
	T. monococcum	36.96 ± 0.45^{de}	5.43 ± 0.35^{c}	14.70	31.00 ± 0.57^{bc}	23.00 ± 0.57^{bc}	2.06 ± 0.01^{d}	0.89 ± 0.01^{b}	2.31 ± 0.01^{c}	2.95 ± 0.01^{d}	0.48 ± 0.01^{bc}
	T. speltoides	25.30 ± 0.62^{b}	3.77 ± 0.21^{ab}	14.89	22.00 ± 0.57^{ab}	20.67 ± 0.33^{b}	2.23 ± 0.01^{de}	0.89 ± 0.01^{b}	2.51 ± 0.01^{d}	3.13 ± 0.02^{de}	0.81 ± 0.02^{e}
200 mM KCl + 500 uM GB	*T. boeoticum*	22.57 ± 0.56^{ab}	4.10 ± 0.10^{b}	18.17	24.33 ± 0.33^{ab}	23.33 ± 0.33^{bc}	2.23 ± 0.01^{de}	0.89 ± 0.01^{b}	2.51 ± 0.01^{d}	3.12 ± 0.01^{de}	0.83 ± 0.03^{ef}
	T. dicoccum	32.10 ± 0.46^{c}	5.10 ± 0.20^{bc}	15.89	29.00 ± 0.57^{b}	21.67 ± 0.66^{b}	1.67 ± 0.01^{c}	0.74 ± 0.01^{ab}	2.26 ± 0.01^{c}	2.40 ± 0.01^{b}	0.43 ± 0.01^{b}
	T. monococcum	31.33 ± 0.68^{c}	4.60 ± 0.20^{b}	14.68	27.67 ± 0.33^{b}	22.67 ± 0.33^{bc}	1.78 ± 0.01^{cd}	0.78 ± 0.01^{ab}	2.28 ± 0.01^{c}	2.56 ± 0.01^{bc}	0.50 ± 0.01^{bc}
	T. speltoides	21.83 ± 1.02^{a}	3.70 ± 0.10^{ab}	16.95	24.00 ± 0.57^{ab}	21.67 ± 0.33^{b}	2.17 ± 0.01^{de}	0.82 ± 0.01^{b}	2.65 ± 0.01^{d}	2.99 ± 0.02^{d}	0.78 ± 0.02^{e}
50 mM NaCl + 50 mM KCl + 500 uM GB	*T. boeoticum*	29.13 ± 0.68^{bc}	4.70 ± 0.26^{bc}	16.13	28.33 ± 0.33^{b}	25.00 ± 0.58^{c}	3.24 ± 0.02^{gh}	1.10 ± 0.01^{f}	2.95 ± 0.02^{e}	4.35 ± 0.02^{i}	0.93 ± 0.02^{f}
	T. dicoccum	20.53 ± 0.18^{a}	5.87 ± 0.31^{cd}	28.57	39.00 ± 0.57^{d}	28.00 ± 0.57^{d}	3.62 ± 0.01^{i}	0.98 ± 0.01^{d}	3.69 ± 0.01^{g}	4.60 ± 0.01^{j}	0.55 ± 0.01^{c}
	T. monococcum	37.73 ± 0.61^{de}	6.13 ± 0.15^{d}	16.25	36.00 ± 0.57^{c}	24.00 ± 0.57^{c}	3.46 ± 0.01^{h}	0.99 ± 0.01^{d}	3.49 ± 0.01^{f}	4.45 ± 0.01^{i}	0.59 ± 0.00^{cd}
	T. speltoides	27.37 ± 0.74^{bc}	4.56 ± 0.21^{b}	16.68	27.67 ± 0.33^{b}	24.67 ± 0.33^{c}	3.14 ± 0.01^{g}	1.08 ± 0.01^{ef}	2.91 ± 0.01^{e}	4.22 ± 0.02^{h}	0.85 ± 0.03^{ef}
100 mM NaCl + 100 mM KCl + 500 uM GB	*T. boeoticum*	25.57 ± 0.85^{b}	4.43 ± 0.20^{b}	17.34	24.33 ± 0.33^{ab}	22.33 ± 0.66^{bc}	2.80 ± 0.01^{f}	1.10 ± 0.01^{f}	2.55 ± 0.01^{d}	3.91 ± 0.01^{g}	0.97 ± 0.03^{f}
	T. dicoccum	37.66 ± 0.56^{de}	5.23 ± 0.15^{c}	13.89	34.00 ± 0.57^{c}	25.33 ± 0.33^{c}	2.58 ± 0.01^{ef}	0.98 ± 0.01^{d}	2.63 ± 0.01^{d}	3.56 ± 0.01^{f}	0.52 ± 0.01^{c}
	T. monococcum	36.40 ± 0.98^{d}	4.96 ± 0.15^{bc}	13.65	35.33 ± 0.33^{c}	26.00 ± 0.57^{cd}	2.78 ± 0.01^{f}	1.01 ± 0.01^{de}	2.75 ± 0.01^{de}	3.80 ± 0.01^{g}	0.56 ± 0.01^{c}
	T. speltoides	25.43 ± 0.65^{b}	4.27 ± 0.15^{b}	16.77	22.00 ± 0.57^{ab}	22.00 ± 0.57^{b}	2.73 ± 0.01^{f}	1.09 ± 0.01^{ef}	2.50 ± 0.01^{d}	3.82 ± 0.02^{g}	0.92 ± 0.02^{f}

* Different letters in the columns indicate significant differences among wheat types ($p<0.05$).

Table S1.4. Enzymatic and non-enzymatic antioxidant defense system responses in roots to individual salt treatments

Salt treatments	***Wheats***	***SOD*** (U mL^{-1} protein)	***CAT*** (U mL^{-1} protein)	***GR*** (U mL^{-1} protein)	***GST*** (U mL^{-1} protein)	***APX*** (U mL^{-1} protein)	***PRO*** (nmol gr^{-1} fw)	***MDA*** (nmol gr^{-1} fw)	***Total Protein*** (µg mL^{-1} protein)
Control	*T. monococcum*	0.857 ± 0.035^{bc}	0.015 ± 0.002^{a}	0.075 ± 0.001^{a}	0.073 ± 0.002^{a}	0.280 ± 0.014^{b}	56.33 ± 2.89^{a}	19.40 ± 0.26^{a}	330.33 ± 12.66^{b}
	T. dicoccum	0.910 ± 0.004^{d}	0.014 ± 0.001^{a}	0.072 ± 0.001^{a}	0.072 ± 0.002^{a}	0.246 ± 0.003^{a}	61.33 ± 0.58^{a}	16.13 ± 0.15^{a}	346.00 ± 7.00^{b}
	T. speltoides	1.119 ± 0.008^{gh}	0.017 ± 0.001^{ab}	0.126 ± 0.002^{cd}	0.144 ± 0.002^{d}	0.313 ± 0.003^{d}	85.00 ± 1.00^{a}	21.67 ± 0.58^{a}	280.67 ± 12.34^{a}
	T. boeoticum	1.005 ± 0.008^{f}	0.017 ± 0.001^{ab}	0.085 ± 0.009^{ab}	0.134 ± 0.002^{c}	0.276 ± 0.002^{b}	73.33 ± 1.15^{a}	20.33 ± 0.58^{a}	308.33 ± 6.50^{a}
30 mM NaCl	*T. monococcum*	0.930 ± 0.007^{de}	0.033 ± 0.002^{c}	0.107 ± 0.003^{bc}	0.099 ± 0.003^{b}	0.327 ± 0.003^{d}	172.31 ± 5.14^{c}	40.33 ± 0.58^{b}	382.67 ± 12.66^{c}
	T. dicoccum	1.023 ± 0.011^{f}	0.033 ± 0.001^{c}	0.107 ± 0.002^{bc}	0.101 ± 0.002^{b}	0.299 ± 0.003^{c}	292.33 ± 1.53^{e}	37.00 ± 1.00^{b}	424.00 ± 10.81^{d}
	T. speltoides	1.148 ± 0.006^{h}	0.049 ± 0.001^{d}	0.218 ± 0.003^{fg}	0.138 ± 0.003^{cd}	0.353 ± 0.002^{e}	335.33 ± 2.52^{g}	54.33 ± 0.58^{bc}	355.00 ± 7.55^{b}
	T. boeoticum	1.086 ± 0.009^{g}	0.045 ± 0.001^{d}	0.187 ± 0.002^{ef}	0.126 ± 0.002^{c}	0.332 ± 0.002^{de}	303.00 ± 2.65^{f}	51.00 ± 1.00^{bc}	347.67 ± 3.05^{b}
30 mM KCl	*T. monococcum*	0.874 ± 0.004^{c}	0.017 ± 0.001^{ab}	0.096 ± 0.002^{b}	0.087 ± 0.002^{ab}	0.309 ± 0.003^{cd}	229.04 ± 1.00^{d}	38.00 ± 1.00^{b}	459.33 ± 7.02^{e}
	T. dicoccum	0.874 ± 0.004^{c}	0.017 ± 0.001^{ab}	0.100 ± 0.002^{b}	0.107 ± 0.002^{b}	0.296 ± 0.002^{c}	244.00 ± 3.00^{d}	36.33 ± 1.53^{b}	472.00 ± 3.61^{e}
	T. speltoides	0.945 ± 0.008^{e}	0.045 ± 0.001^{d}	0.195 ± 0.004^{f}	0.125 ± 0.003^{c}	0.345 ± 0.001^{e}	293.00 ± 2.65^{e}	45.33 ± 1.53^{b}	419.00 ± 8.88^{d}
	T. boeoticum	0.919 ± 0.008^{d}	0.043 ± 0.001^{cd}	0.174 ± 0.002^{e}	0.114 ± 0.003^{bc}	0.323 ± 0.002^{d}	280.33 ± 3.51^{e}	42.00 ± 1.00^{b}	420.67 ± 9.50^{d}
50 mM NaCl	*T. monococcum*	0.974 ± 0.002^{ef}	0.040 ± 0.002^{cd}	0.140 ± 0.002^{d}	0.111 ± 0.002^{bc}	0.352 ± 0.004^{e}	265.05 ± 2.00^{de}	57.33 ± 1.15b^{c}	416.67 ± 6.40^{d}
	T. dicoccum	0.961 ± 0.007^{e}	0.038 ± 0.001^{cd}	0.145 ± 0.001^{de}	0.144 ± 0.002^{d}	0.335 ± 0.002^{de}	301.67 ± 3.06^{f}	51.00 ± 1.00^{bc}	402.00 ± 3.00^{d}
	T. speltoides	1.118 ± 0.006^{gh}	0.055 ± 0.002^{e}	0.296 ± 0.006^{i}	0.167 ± 0.002^{e}	0.386 ± 0.002^{g}	403.33 ± 3.21^{h}	74.33 ± 0.58^{c}	366.00 ± 9.16^{c}
	T. boeoticum	0.994 ± 0.004^{f}	0.053 ± 0.002^{de}	0.264 ± 0.001^{h}	0.147 ± 0.002^{d}	0.374 ± 0.002^{f}	385.50 ± 0.71^{h}	68.50 ± 0.71^{c}	385.67 ± 8.02^{c}
50 mM KCl	*T. monococcum*	0.905 ± 0.004^{cd}	0.022 ± 0.002^{b}	0.128 ± 0.002^{cd}	0.101 ± 0.003^{b}	0.316 ± 0.002^{d}	357.75 ± 2.52^{g}	56.00 ± 2.00^{bc}	501.00 ± 3.61^{f}
	T. dicoccum	0.919 ± 0.005^{d}	0.022 ± 0.001^{b}	0.123 ± 0.001^{cd}	0.125 ± 0.001^{c}	0.304 ± 0.003^{cd}	394.33 ± 3.51^{h}	47.67 ± 0.58^{b}	512.33 ± 9.61^{f}
	T. speltoides	1.125 ± 0.011^{gh}	0.055 ± 0.001^{e}	0.258 ± 0.004^{h}	0.144 ± 0.001^{d}	0.368 ± 0.003^{f}	476.67 ± 3.79^{ij}	69.67 ± 1.15^{c}	468.67 ± 6.11^{e}
	T. boeoticum	1.098 ± 0.008^{g}	0.053 ± 0.002^{de}	0.232 ± 0.005^{g}	0.137 ± 0.002^{cd}	0.358 ± 0.004^{ef}	443.33 ± 3.21^{i}	61.67 ± 1.53^{bc}	483.33 ± 7.09^{f}
100 mM NaCl	*T. monococcum*	0.957 ± 0.003^{e}	0.033 ± 0.001^{c}	0.138 ± 0.003^{d}	0.101 ± 0.003^{b}	0.325 ± 0.003^{d}	455.27 ± 2.08^{i}	165.33 ± 2.52^{d}	430.67 ± 5.57^{e}
	T. dicoccum	0.915 ± 0.004^{d}	0.033 ± 0.001^{c}	0.133 ± 0.001^{d}	0.138 ± 0.003^{cd}	0.305 ± 0.003^{cd}	383.33 ± 2.52^{h}	152.67 ± 1.53^{d}	399.00 ± 3.00^{d}
	T. speltoides	1.124 ± 0.012^{gh}	0.048 ± 0.001^{d}	0.312 ± 0.006^{i}	0.152 ± 0.002^{d}	0.341 ± 0.003^{e}	491.33 ± 6.35^{j}	207.67 ± 3.51^{e}	363.00 ± 6.55^{c}
	T. boeoticum	1.066 ± 0.012^{g}	0.046 ± 0.001^{d}	0.303 ± 0.001^{i}	0.140 ± 0.002^{cd}	0.322 ± 0.003^{d}	438.67 ± 2.52^{i}	192.33 ± 1.53^{e}	374.33 ± 3.51^{c}
100 mM KCl	*T. monococcum*	0.921 ± 0.002^{d}	0.016 ± 0.001^{a}	0.120 ± 0.002^{c}	0.094 ± 0.001^{b}	0.304 ± 0.002^{cd}	451.32 ± 1.15^{i}	158.33 ± 0.58^{d}	475.33 ± 4.16^{e}
	T. dicoccum	0.887 ± 0.020^{c}	0.014 ± 0.001^{a}	0.118 ± 0.004^{c}	0.120 ± 0.002^{bc}	0.277 ± 0.003^{b}	495.33 ± 2.08^{j}	149.67 ± 1.53^{d}	480.67 ± 6.03^{f}
	T. speltoides	1.160 ± 0.032^{h}	0.049 ± 0.002^{d}	0.238 ± 0.003^{g}	0.135 ± 0.001^{c}	0.347 ± 0.003^{e}	561.33 ± 7.02^{l}	195.33 ± 1.53^{e}	459.33 ± 3.51^{e}
	T. boeoticum	1.145 ± 0.053^{h}	0.046 ± 0.001^{d}	0.204 ± 0.003^{f}	0.130 ± 0.002^{c}	0.321 ± 0.004^{d}	537.67 ± 6.03^{k}	190.67 ± 1.53^{e}	460.67 ± 7.63^{e}
150 mM NaCl	*T. monococcum*	0.920 ± 0.003^{d}	0.022 ± 0.002^{b}	0.112 ± 0.002^{c}	0.084 ± 0.002^{ab}	0.299 ± 0.003^{c}	231.31 ± 1.53^{d}	267.00 ± 2.65^{g}	425.33 ± 8.17^{d}
	T. dicoccum	0.864 ± 0.004^{bc}	0.024 ± 0.001^{b}	0.108 ± 0.003^{bc}	0.088 ± 0.003^{ab}	0.276 ± 0.002^{b}	253.33 ± 2.08^{d}	242.67 ± 1.53^{f}	384.33 ± 9.07^{c}
	T. speltoides	1.009 ± 0.006^{f}	0.042 ± 0.001^{cd}	0.265 ± 0.003^{h}	0.444 ± 0.079^{g}	0.314 ± 0.002^{d}	275.33 ± 3.06^{e}	318.33 ± 2.52^{h}	333.00 ± 7.55^{b}
	T. boeoticum	0.970 ± 0.007^{ef}	0.040 ± 0.001^{cd}	0.212 ± 0.003^{fg}	0.106 ± 0.002^{b}	0.306 ± 0.002^{cd}	268.00 ± 3.00^{de}	304.00 ± 1.73^{h}	352.33 ± 5.50^{b}
150 mM KCl	*T. monococcum*	0.901 ± 0.007^{cd}	0.014 ± 0.002^{a}	0.116 ± 0.003^{c}	0.082 ± 0.006^{ab}	0.298 ± 0.005^{c}	344.05 ± 2.65^{g}	261.67 ± 0.58^{g}	466.00 ± 4.00^{e}
	T. dicoccum	0.858 ± 0.004^{bc}	0.012 ± 0.001^{a}	0.113 ± 0.002^{c}	0.117 ± 0.002^{bc}	0.273 ± 0.002^{b}	365.33 ± 3.21^{gh}	243.33 ± 2.08^{f}	453.33 ± 6.11^{e}
	T. speltoides	1.086 ± 0.004^{g}	0.042 ± 0.001^{cd}	0.192 ± 0.005^{f}	0.126 ± 0.002^{c}	0.332 ± 0.002^{de}	394.33 ± 3.51^{h}	339.00 ± 3.61^{hi}	445.67 ± 4.72^{d}
	T. boeoticum	1.016 ± 0.010^{f}	0.038 ± 0.001^{cd}	0.171 ± 0.003^{e}	0.116 ± 0.002^{bc}	0.320 ± 0.004^{d}	387.67 ± 3.06^{h}	324.00 ± 2.65^{h}	436.00 ± 3.46^{d}
200 mM NaCl	*T. monococcum*	0.830 ± 0.005^{b}	0.012 ± 0.002^{a}	0.076 ± 0.005^{a}	0.075 ± 0.001^{a}	0.267 ± 0.004^{b}	127.78 ± 2.52^{b}	376.67 ± 1.53i	353.00 ± 10.82^{b}
	T. dicoccum	0.797 ± 0.006^{a}	0.014 ± 0.001^{a}	0.073 ± 0.003^{a}	0.081 ± 0.001^{ab}	0.252 ± 0.002^{a}	138.33 ± 3.51^{b}	343.67 ± 2.52h^{i}	344.00 ± 3.00^{b}
	T. speltoides	0.911 ± 0.005^{d}	0.034 ± 0.001^{c}	0.145 ± 0.002^{de}	0.098 ± 0.002^{b}	0.288 ± 0.002^{bc}	159.67 ± 2.52^{c}	404.00 ± 2.00^{j}	307.67 ± 7.37^{a}
	T. boeoticum	0.881 ± 0.004^{c}	0.031 ± 0.001^{c}	0.116 ± 0.002^{c}	0.090 ± 0.002^{ab}	0.276 ± 0.002^{b}	154.00 ± 1.00^{bc}	392.33 ± 1.53^{j}	310.33 ± 4.16^{a}
200 mM KCl	*T. monococcum*	0.840 ± 0.002^{b}	0.015 ± 0.001^{a}	0.080 ± 0.002^{a}	0.074 ± 0.002^{a}	0.258 ± 0.003^{ab}	131.31 ± 1.15^{b}	365.67 ± 0.58^{i}	428.67 ± 8.33^{d}
	T. dicoccum	0.808 ± 0.007^{a}	0.014 ± 0.001^{a}	0.077 ± 0.002^{a}	0.105 ± 0.002^{b}	0.244 ± 0.003^{a}	142.00 ± 2.00^{b}	354.67 ± 3.06^{i}	427.33 ± 4.62^{d}
	T. speltoides	0.991 ± 0.006^{f}	0.036 ± 0.001^{c}	0.160 ± 0.002^{e}	0.111 ± 0.002^{bc}	0.308 ± 0.003^{cd}	152.67 ± 1.53^{bc}	417.00 ± 4.36^{j}	394.67 ± 7.02^{c}
	T. boeoticum	0.911 ± 0.008^{d}	0.035 ± 0.001^{c}	0.120 ± 0.006^{c}	0.098 ± 0.002^{b}	0.294 ± 0.003^{c}	138.33 ± 2.52^{b}	403.00 ± 2.65^{j}	390.00 ±7.81^{a}

* Different letters in the columns indicate significant differences among wheat types ($p<0.05$).

Table S1.5. Enzymatic and non-enzymatic antioxidant defense system responses in roots to combined salt treatments

Salt treatments	***Wheats***	***SOD*** (U mL^{-1} protein)	***CAT*** (U mL^{-1} protein)	***GR*** (U mL^{-1} protein)	***GST*** (U mL^{-1} protein)	***APX*** (U mL^{-1} protein)	***PRO*** (nmol gr^{-1} fw)	***MDA*** (nmol gr^{-1} fw)	***Total Protein*** (µg mL^{-1} protein)
Control	*T. monococcum*	0.857 ± 0.035^{bc}	0.015 ± 0.002^{a}	0.075 ± 0.001^{a}	0.073 ± 0.002^{a}	0.280 ± 0.014^{b}	56.33 ± 2.89^{a}	19.40 ± 0.26^{a}	330.33 ± 12.66^{b}
	T. dicoccum	0.910 ± 0.004^{d}	0.014 ± 0.001^{a}	0.072 ± 0.001^{a}	0.072 ± 0.002^{a}	0.246 ± 0.003^{a}	61.33 ± 0.58^{a}	16.13 ± 0.15^{a}	346.00 ± 7.00^{b}
	T. speltoides	1.119 ± 0.008^{gh}	0.017 ± 0.001^{ab}	0.126 ± 0.002^{cd}	0.144 ± 0.002^{d}	0.313 ± 0.003^{d}	85.00 ± 1.00^{a}	21.67 ± 0.58^{a}	280.67 ± 12.34^{a}
	T. boeoticum	1.005 ± 0.008^{f}	0.017 ± 0.001^{ab}	0.085 ± 0.009^{ab}	0.134 ± 0.002^{c}	0.276 ± 0.002^{b}	73.33 ± 1.15^{a}	20.33 ± 0.58^{a}	308.33 ± 6.50^{a}
50 mM NaCl + 50 mM KCl	*T. monococcum*	1.057 ± 0.001^{g}	0.030 ± 0.002^{c}	0.144 ± 0.002^{de}	0.121 ± 0.006^{bc}	0.337 ± 0.011^{de}	570.72 ± 4.16^{l}	53.00 ± 1.00^{bc}	558.00 ± 8.72^{g}
	T. dicoccum	1.013 ± 0.012^{f}	0.030 ± 0.001^{c}	0.145 ± 0.003^{de}	0.142 ± 0.002^{cd}	0.307 ± 0.003^{cd}	608.33 ± 6.51^{m}	46.00 ± 1.00^{b}	561.00 ± 7.00^{g}
	T. speltoides	1.34 ± 0.014^{i}	0.045 ± 0.001^{d}	0.282 ± 0.007^{hi}	0.165 ± 0.002^{e}	0.335 ± 0.003^{de}	652.00 ± 4.58^{o}	63.33 ± 1.53^{bc}	499.33 ± 11.93^{f}
	T. boeoticum	1.305 ± 0.010^{i}	0.041 ± 0.001^{cd}	0.269 ± 0.005^{hi}	0.150 ± 0.002^{d}	0.325 ± 0.004^{d}	631.67 ± 8.33^{n}	56.00 ± 2.00^{bc}	502.33 ± 4.72^{f}
100 mM NaCl + 100 mM KCl	*T. monococcum*	0.872 ± 0.002^{c}	0.017 ± 0.001^{ab}	0.083 ± 0.001^{ab}	0.076 ± 0.001^{a}	0.294 ± 0.002^{c}	603.33 ± 5.72^{m}	163.67 ± 1.53^{d}	494.33 ± 8.02^{f}
	T. dicoccum	0.797 ± 0.005^{a}	0.016 ± 0.001^{a}	0.089 ± 0.003^{ab}	0.094 ± 0.001^{b}	0.282 ± 0.002^{bc}	575.33 ± 5.13^{lm}	142.67 ± 1.53^{d}	507.33 ± 4.73^{f}
	T. speltoides	0.991 ± 0.003^{f}	0.039 ± 0.001^{cd}	0.201 ± 0.006^{f}	0.106 ± 0.002^{b}	0.294 ± 0.005^{c}	605.00 ± 3.61^{m}	198.33 ± 2.52^{e}	480.00 ± 7.93^{e}
	T. boeoticum	0.910 ± 0.008^{d}	0.035 ± 0.001^{c}	0.183 ± 0.005^{ef}	0.100 ± 0.002^{b}	0.312 ± 0.003^{d}	585.67 ± 5.51^{lm}	184.00 ± 3.00^{e}	477.00 ± 7.81^{e}
150 mM NaCl + 500 uM GB	*T. monococcum*	0.974 ± 0.004^{ef}	0.028 ± 0.001^{bc}	0.123 ± 0.003^{cd}	0.103 ± 0.002^{b}	0.371 ± 0.003^{f}	374.04 ± 2.65^{h}	251.00 ± 3.00^{fg}	544.33 ± 3.51^{g}
	T. dicoccum	0.886 ± 0.002^{c}	0.029 ± 0.001^{bc}	0.120 ± 0.002^{c}	0.111 ± 0.003^{bc}	0.318 ± 0.002^{d}	398.67 ± 3.06^{h}	224.00 ± 2.00^{ef}	563.67 ± 4.51^{h}
	T. speltoides	1.044 ± 0.003^{g}	0.052 ± 0.001^{de}	0.283 ± 0.002^{hi}	0.123 ± 0.002^{c}	0.346 ± 0.003^{e}	442.67 ± 2.08^{i}	287.33 ± 1.53^{gh}	514.00 ± 6.55^{f}
	T. boeoticum	1.027 ± 0.002^{f}	0.047 ± 0.002^{d}	0.246 ± 0.004^{gh}	0.123 ± 0.003^{c}	0.328 ± 0.005^{de}	429.33 ± 1.53^{i}	273.67 ± 3.79^{g}	514.33 ± 5.68^{f}
150 mM KCl + 500 uM GB	*T. monococcum*	0.957 ± 0.004^{e}	0.019 ± 0.002^{ab}	0.151 ± 0.003^{de}	0.112 ± 0.004^{bc}	0.407 ± 0.006^{h}	421.33 ± 5.51^{hi}	223.67 ± 1.53^{ef}	533.33 ± 4.16^{g}
	T. dicoccum	0.907 ± 0.004^{cd}	0.017 ± 0.002^{ab}	0.144 ± 0.002^{de}	0.145 ± 0.001^{d}	0.392 ± 0.004^{g}	449.00 ± 5.29^{i}	212.00 ± 3.00^{e}	540.33 ± 3.21^{g}
	T. speltoides	1.109 ± 0.006^{gh}	0.053 ± 0.001^{de}	0.244 ± 0.005^{gh}	0.160 ± 0.003^{de}	0.491 ± 0.009^{j}	495.67 ± 3.51^{j}	265.67 ± 3.06^{g}	563.67 ± 4.72^{h}
	T. boeoticum	1.081 ± 0.004^{g}	0.047 ± 0.002^{d}	0.208 ± 0.004^{fg}	0.146 ± 0.003^{d}	0.433 ± 0.006^{i}	487.67 ± 3.06^{j}	243.00 ± 2.65^{f}	546.67 ± 4.51^{g}
200 mM NaCl + 500 uM GB	*T. monococcum*	0.884 ± 0.003^{c}	0.019 ± 0.001^{ab}	0.095 ± 0.001^{b}	0.096 ± 0.002^{b}	0.355 ± 0.004^{e}	233.71 ± 3.79^{d}	313.00 ± 2.65^{h}	508.33 ± 5.13^{f}
	T. dicoccum	0.865 ± 0.003^{bc}	0.020 ± 0.001^{ab}	0.090 ± 0.003^{ab}	0.103 ± 0.002^{b}	0.294 ± 0.007^{c}	249.33 ± 4.51^{d}	297.67 ± 1.53^{h}	524.33 ± 3.51^{g}
	T. speltoides	0.969 ± 0.005^{ef}	0.044 ± 0.001^{d}	0.164 ± 0.003^{e}	0.113 ± 0.003^{bc}	0.320 ± 0.006^{d}	292.33 ± 3.21^{e}	335.00 ± 2.65^{hi}	499.33 ± 4.16^{f}
	T. boeoticum	0.899 ± 0.005^{cd}	0.016 ± 0.002^{a}	0.142 ± 0.004^{de}	0.114 ± 0.001^{bc}	0.301 ± 0.006^{c}	290.00 ± 2.65^{e}	322.33 ± 1.53^{h}	495.67 ± 7.76^{f}
200 mM KCl + 500 uM GB	*T. monococcum*	0.904 ± 0.002^{cd}	0.024 ± 0.001^{b}	0.097 ± 0.002^{b}	0.105 ± 0.003^{b}	0.383 ± 0.003^{g}	233.33 ± 2.08^{d}	278.67 ± 2.08^{g}	590.00 ± 4.00^{h}
	T. dicoccum	0.884 ± 0.006^{c}	0.023 ± 0.001^{b}	0.091 ± 0.002^{ab}	0.125 ± 0.004^{c}	0.363 ± 0.005^{ef}	243.33 ± 2.08^{d}	268.67 ± 2.08^{g}	581.00 ± 9.75^{h}
	T. speltoides	1.067 ± 0.005^{g}	0.045 ± 0.001^{d}	0.192 ± 0.005^{f}	0.137 ± 0.002^{cd}	0.391 ± 0.005^{g}	275.67 ± 2.52^{e}	335.67 ± 2.52^{hi}	628.33 ± 1.52^{i}
	T. boeoticum	1.001 ± 0.007^{f}	0.048 ± 0.001^{d}	0.174 ± 0.003^{e}	0.117 ± 0.005^{bc}	0.390 ± 0.006^{g}	231.33 ± 3.51^{d}	316.67 ± 2.08^{h}	604.00 ± 2.64^{i}
50 mM NaCl + 50 mM KCl + 500 uM GB	*T. monococcum*	1.105 ± 0.003^{gh}	0.040 ± 0.001^{cd}	0.185 ± 0.003^{ef}	0.152 ± 0.003^{d}	0.408 ± 0.005^{h}	663.71 ± 6.43^{o}	40.33 ± 0.58^{b}	583.33 ± 2.08^{h}
	T. dicoccum	1.088 ± 0.006^{g}	0.038 ± 0.001^{cd}	0.197 ± 0.003^{f}	0.192 ± 0.004^{f}	0.396 ± 0.004^{g}	754.00 ± 8.89^{p}	38.67 ± 1.15^{b}	594.00 ± 3.61^{h}
	T. speltoides	1.423 ± 0.010^{j}	0.055 ± 0.001^{e}	0.331 ± 0.006^{j}	0.185 ± 0.001^{f}	0.434 ± 0.003^{i}	799.00 ± 8.89^{r}	42.67 ± 1.53^{b}	603.67 ± 1.52^{i}
	T. boeoticum	1.393 ± 0.011^{ij}	0.052 ± 0.001^{de}	0.327 ± 0.002^{j}	0.187 ± 0.002^{f}	0.426 ± 0.002^{i}	777.00 ± 9.54^{r}	40.67 ± 1.53^{b}	608.00 ± 6.00^{i}
100 mM NaCl + 100 mM KCl + 500 uM GB	*T. monococcum*	0.936 ± 0.004^{de}	0.031 ± 0.001^{c}	0.090 ± 0.002^{ab}	0.113 ± 0.003^{bc}	0.372 ± 0.007^{f}	704.33 ± 8.50^{op}	98.33 ± 2.52^{cd}	672.33 ± 3.51^{k}
	T. dicoccum	0.879 ± 0.004^{c}	0.029 ± 0.001^{bc}	0.097 ± 0.002^{b}	0.135 ± 0.003^{c}	0.354 ± 0.003^{e}	781.67 ± 11.59^{pr}	94.33 ± 1.53^{cd}	673.67 ± 4.51^{k}
	T. speltoides	0.741 ± 0.058^{a}	0.051 ± 0.001^{de}	0.244 ± 0.004^{gh}	0.143 ± 0.002^{d}	0.384 ± 0.003^{g}	807.67 ± 6.66^{r}	111.67 ± 3.51^{cd}	682.00 ± 2.00^{k}
	T. boeoticum	1.031 ± 0.004^{f}	0.051 ± 0.001^{de}	0.208 ± 0.006^{fg}	0.121 ± 0.003^{bc}	0.381 ± 0.004^{g}	797.00 ± 3.61^{r}	109.00 ± 3.00^{cd}	643.67 ± 2.30^{j}
TOTAL	*T. monococcum*	0.926 ± 0.068^{de}	0.024 ± 0.009^{b}	0.114 ± 0.029^{c}	0.098 ± 0.019^{b}	0.330 ± 0.044^{de}	348.75 ± 186.09^{g}	169.86 ± 117.79^{d}	481.74 ± 85.91^{f}
	T. dicoccum	0.902 ± 0.075^{d}	0.023 ± 0.009^{b}	0.113 ± 0.031^{c}	0.118 ± 0.027^{bc}	0.306 ± 0.044^{cd}	375.32 ± 195.15^{gh}	157.83 ± 111.25^{d}	485.46 ± 90.92^{f}
	T. speltoides	1.075 ± 0.068^{g}	0.045 ± 0.009^{d}	0.229 ± 0.058^{g}	0.153 ± 0.071^{d}	0.352 ± 0.051^{e}	420.95 ± 200.83^{hi}	199.28 ± 133.30^{e}	454.89 ± 110.31^{e}
	T. boeoticum	1.040 ± 0.132^{fg}	0.049 ± 0.003^{d}	0.200 ± 0.063^{f}	0.126 ± 0.022^{c}	0.336 ± 0.045^{de}	402.38 ± 200.44^{h}	191.38 ± 128.73^{e}	456.95 ± 98.83^{e}

* Different letters in the columns indicate significant differences among wheat types ($p<0.05$).

Table S1.6. Enzymatic and non-enzymatic antioxidant defense system responses in shoot to individual salt treatments

Salt treatments	***Wheats***	***SOD*** (U mL^{-1} protein)	***CAT*** (U mL^{-1} protein)	***GR*** (U mL^{-1} protein)	***GST*** (U mL^{-1} protein)	***APX*** (U mL^{-1} protein)	***PRO*** (nmol gr^{-1} fw)	***MDA*** (nmol gr^{-1} fw)	***Total Protein*** (µg mL^{-1} protein)
Control	*T. monococcum*	0.743 ± 0.035^{b}	0.024 ± 0.009^{bc}	0.081 ± 0.001^{ab}	0.081 ± 0.003^{b}	0.294 ± 0.007^{bc}	50.83 ± 1.27^{a}	17.23 ± 0.29^{a}	462.67 ± 15.01^{cd}
	T. dicoccum	0.783 ± 0.008^{bc}	0.011 ± 0.001^{a}	0.077 ± 0.001^{ab}	0.078 ± 0.001^{ab}	0.272 ± 0.002^{ab}	57.33 ± 1.15^{a}	16.00 ± 1.00^{a}	490.67 ± 13.32^{cd}
	T. speltoides	0.884 ± 0.005^{de}	0.015 ± 0.001^{ab}	0.114 ± 0.002^{d}	0.114 ± 0.002^{cd}	0.323 ± 0.002^{cd}	75.33 ± 1.53^{ab}	18.67 ± 0.58^{a}	413.67 ± 11.59^{b}
	T. boeoticum	0.821 ± 0.008^{cd}	0.016 ± 0.001^{ab}	0.096 ± 0.002^{c}	0.108 ± 0.002^{c}	0.310 ± 0.002^{c}	71.00 ± 1.00^{ab}	17.33 ± 0.58^{a}	433.67 ± 8.73^{ab}
30 mM NaCl	*T. monococcum*	0.814 ± 0.002^{cd}	0.012 ± 0.001^{a}	0.096 ± 0.002^{c}	0.104 ± 0.002^{c}	0.328 ± 0.003^{d}	233.33 ± 1.53^{de}	30.67 ± 1.15^{ab}	500.67 ± 17.24^{e}
	T. dicoccum	0.873 ± 0.003^{de}	0.020 ± 0.001^{b}	0.102 ± 0.004^{c}	0.104 ± 0.006^{c}	0.309 ± 0.006^{c}	283.67 ± 3.79^{ef}	29.00 ± 1.00^{ab}	517.00 ± 17.52^{de}
	T. speltoides	1.039 ± 0.013^{h}	0.028 ± 0.006^{c}	0.150 ± 0.002^{ef}	0.127 ± 0.002^{de}	0.352 ± 0.005^{e}	228.33 ± 2.52^{d}	41.67 ± 0.58^{b}	428.67 ± 8.32^{bc}
	T. boeoticum	0.997 ± 0.011^{g}	0.031 ± 0.001^{c}	0.142 ± 0.002^{e}	0.124 ± 0.002^{d}	0.341 ± 0.002^{d}	293.00 ± 2.65^{f}	35.67 ± 0.58^{b}	458.33 ± 4.50^{cd}
30 mM KCl	*T. monococcum*	0.793 ± 0.003^{c}	0.012 ± 0.002^{a}	0.086 ± 0.003^{b}	0.081 ± 0.002^{b}	0.293 ± 0.002^{bc}	220.33 ± 1.53^{d}	33.67 ± 0.58^{b}	485.67 ± 8.02^{d}
	T. dicoccum	0.772 ± 0.003^{bc}	0.012 ± 0.001^{a}	0.086 ± 0.002^{b}	0.090 ± 0.002^{b}	0.280 ± 0.002^{b}	243.33 ± 2.08^{e}	30.67 ± 0.58^{ab}	479.33 ± 6.11^{d}
	T. speltoides	0.821 ± 0.006^{cd}	0.019 ± 0.001^{b}	0.140 ± 0.002^{e}	0.100 ± 0.002^{c}	0.326 ± 0.002^{d}	276.00 ± 2.65^{ef}	35.00 ± 1.00^{b}	457.33 ± 7.76^{cd}
	T. boeoticum	0.794 ± 0.009^{c}	0.017 ± 0.001^{ab}	0.126 ± 0.002^{de}	0.100 ± 0.002^{c}	0.318 ± 0.004^{cd}	265.67 ± 5.03^{e}	32.33 ± 0.58^{b}	446.33 ±10.40^{c}
50 mM NaCl	*T. monococcum*	0.845 ± 0.003^{d}	0.021 ± 0.001^{b}	0.105 ± 0.001^{cd}	0.116 ± 0.002^{d}	0.346 ± 0.002^{de}	251.67 ± 4.04^{e}	37.00 ± 1.00^{b}	532.00 ±9.60^{f}
	T. dicoccum	0.819 ± 0.004^{cd}	0.025 ± 0.001^{bc}	0.110 ± 0.003^{d}	0.156 ± 0.002^{f}	0.322 ± 0.002^{cd}	306.67 ± 1.53^{f}	32.33 ± 0.58^{b}	528.33 ± 9.29^{f}
	T. speltoides	0.877 ± 0.003^{de}	0.034 ± 0.001^{d}	0.197 ± 0.002^{g}	0.171 ± 0.001^{g}	0.380 ± 0.002^{f}	363.33 ± 1.53^{gh}	47.00 ± 1.00^{bc}	470.67 ± 2.51^{d}
	T. boeoticum	0.858 ± 0.005^{d}	0.032 ± 0.001^{cd}	0.347 ± 0.026^{j}	0.156 ± 0.017^{f}	0.354 ± 0.003^{e}	323.33 ± 2.08^{f}	44.67 ± 0.58^{b}	477.33 ± 4.72^{d}
50 mM KCl	*T. monococcum*	0.809 ± 0.003^{c}	0.014 ± 0.001^{a}	0.099 ± 0.004^{c}	0.088 ± 0.004^{b}	0.314 ± 0.002^{c}	356.67 ± 1.15^{g}	39.67 ± 0.58^{b}	536.00 ± 9.16^{f}
	T. dicoccum	0.781 ± 0.003^{bc}	0.017 ± 0.001^{ab}	0.094 ± 0.002^{bc}	0.109 ± 0.003^{c}	0.302 ± 0.004^{c}	384.00 ± 2.00^{h}	36.00 ± 1.00^{b}	532.00 ± 8.72^{f}
	T. speltoides	0.960 ± 0.007^{f}	0.024 ± 0.002^{bc}	0.175 ± 0.004^{fg}	0.116 ± 0.002^{d}	0.344 ± 0.003^{de}	412.67 ± 2.08^{hi}	57.33 ± 0.58^{c}	510.00 ± 5.29^{e}
	T. boeoticum	0.659 ± 0.049^{a}	0.022 ± 0.001^{b}	0.165 ± 0.003^{f}	0.113 ± 0.004^{cd}	0.341 ± 0.004^{d}	400.67 ± 3.51^{h}	50.33 ± 0.58^{bc}	509.67 ± 2.51^{e}
100 mM NaCl	*T. monococcum*	0.811 ± 0.002^{cd}	0.023 ± 0.001^{bc}	0.099 ± 0.003^{c}	0.100 ± 0.003^{c}	0.318 ± 0.003^{cd}	338.33 ± 1.53^{g}	145.33 ± 1.53^{e}	551.67 ± 3.21^{fg}
	T. dicoccum	0.774 ± 0.012^{bc}	0.022 ± 0.002^{b}	0.090 ± 0.002^{b}	0.129 ± 0.003^{de}	0.303 ± 0.002^{c}	376.67 ± 2.08^{gh}	125.33 ± 1.53^{e}	536.33 ± 4.51^{f}
	T. speltoides	0.885 ± 0.013^{de}	0.031 ± 0.001^{c}	0.208 ± 0.003^{h}	0.145 ± 0.001^{e}	0.354 ± 0.002^{e}	421.67 ± 3.51^{i}	186.00 ± 3.00^{f}	498.00 ± 8.88^{e}
	T. boeoticum	0.871 ± 0.002^{de}	0.031 ± 0.001^{c}	0.183 ± 0.002^{fg}	0.136 ± 0.002^{e}	0.334 ± 0.003^{d}	406.33 ± 3.79^{hi}	175.33 ± 1.53^{f}	502.33 ± 5.50^{e}
100 mM KCl	*T. monococcum*	0.816 ± 0.002^{cd}	0.017 ± 0.001^{ab}	0.096 ± 0.002^{c}	0.081 ± 0.001^{b}	0.314 ± 0.002^{c}	459.33 ± 3.06^{j}	140.33 ± 0.58^{e}	532.67 ± 13.61^{f}
	T. dicoccum	0.794 ± 0.009^{c}	0.014 ± 0.001^{a}	0.091 ± 0.003^{bc}	0.100 ± 0.002^{c}	0.301 ± 0.005^{c}	471.33 ± 3.51^{j}	129.67 ± 1.53^{e}	514.67 ± 11.01^{ef}
	T. speltoides	1.025 ± 0.010^{h}	0.021 ± 0.001^{b}	0.156 ± 0.002^{f}	0.105 ± 0.002^{c}	0.343 ± 0.003^{de}	510.67 ± 4.16^{k}	175.00 ± 1.00^{f}	490.33 ± 13.31^{de}
	T. boeoticum	0.981 ± 0.004^{g}	0.021 ± 0.001^{b}	0.134 ± 0.002^{e}	0.103 ± 0.001^{c}	0.329 ± 0.004^{d}	505.00 ± 3.61^{k}	163.33 ± 1.53^{f}	498.33 ± 11.93^{e}
150 mM NaCl	*T. monococcum*	0.804 ± 0.002^{c}	0.019 ± 0.001^{b}	0.093 ± 0.001^{bc}	0.092 ± 0.002^{bc}	0.286 ± 0.003^{b}	233.00 ± 1.00^{de}	137.67 ± 6.34^{e}	558.00 ± 12.72^{g}
	T. dicoccum	0.774 ± 0.004^{bc}	0.017 ± 0.001^{ab}	0.091 ± 0.003^{bc}	0.114 ± 0.002^{cd}	0.271 ± 0.003^{ab}	271.00 ± 3.00^{ef}	250.33 ± 4.04^{g}	543.00 ± 1.72^{f}
	T. speltoides	0.894 ± 0.004^{e}	0.025 ± 0.001^{bc}	0.159 ± 0.003^{f}	0.122 ± 0.002^{d}	0.329 ± 0.002^{d}	311.67 ± 4.04^{f}	295.67 ± 2.08^{h}	508.00 ± 6.00^{e}
	T. boeoticum	0.856 ± 0.011^{d}	0.023 ± 0.001^{bc}	0.134 ± 0.002^{e}	0.119 ± 0.002^{d}	0.322 ± 0.002^{cd}	303.33 ± 2.08^{f}	286.33 ± 1.53^{h}	496.33 ± 7.50^{e}
150 mM KCl	*T. monococcum*	0.800 ± 0.005^{c}	0.016 ± 0.001^{ab}	0.086 ± 0.002^{b}	0.077 ± 0.002^{ab}	0.304 ± 0.007^{c}	350.00 ± 2.00^{g}	345.33 ± 1.53^{i}	489.33 ± 6.11^{de}
	T. dicoccum	0.772 ± 0.004^{bc}	0.011 ± 0.001^{a}	0.084 ± 0.002^{b}	0.092 ± 0.002^{bc}	0.292 ± 0.002^{bc}	374.00 ± 3.61^{gh}	318.33 ± 2.52^{i}	488.67 ± 2.52^{de}
	T. speltoides	1.000 ± 0.006^{g}	0.017 ± 0.001^{ab}	0.125 ± 0.003^{de}	0.095 ± 0.001^{bc}	0.319 ± 0.003^{cd}	411.33 ± 3.06^{hi}	385.67 ± 3.21^{j}	454.67 ± 8.50^{c}
	T. boeoticum	0.923 ± 0.008^{e}	0.038 ± 0.003^{d}	0.121 ± 0.003^{d}	0.090 ± 0.002^{b}	0.313 ± 0.002^{c}	381.33 ± 4.51^{h}	373.67 ± 2.52^{ij}	459.67 ± 4.72^{cd}
200 mM NaCl	*T. monococcum*	0.747 ± 0.064^{b}	0.016 ± 0.002^{ab}	0.072 ± 0.002^{a}	0.068 ± 0.003^{a}	0.275 ± 0.006^{b}	104.67 ± 0.58^{b}	378.67 ± 0.58^{j}	489.00 ± 5.57^{de}
	T. dicoccum	0.769 ± 0.008^{bc}	0.012 ± 0.001^{a}	0.071 ± 0.002^{a}	0.082 ± 0.002^{b}	0.250 ± 0.002^{a}	121.00 ± 1.00^{b}	368.33 ± 2.52^{ij}	484.33 ± 2.08^{d}
	T. speltoides	0.881 ± 0.007^{de}	0.022 ± 0.001^{b}	0.124 ± 0.004^{de}	0.087 ± 0.002^{b}	0.304 ± 0.003^{c}	143.67 ± 1.53^{c}	422.67 ± 1.53^{k}	432.67 ± 5.50^{bc}
	T. boeoticum	0.854 ± 0.008^{d}	0.022 ± 0.001^{b}	0.108 ± 0.004^{cd}	0.083 ± 0.002^{b}	0.286 ± 0.002^{b}	101.67 ± 6.04^{b}	409.00 ± 2.65^{jk}	438.00 ± 6.08^{c}
200 mM KCl	*T. monococcum*	0.708 ± 0.003^{a}	0.012 ± 0.001^{a}	0.072 ± 0.002^{a}	0.074 ± 0.001^{ab}	0.266 ± 0.002^{ab}	105.67 ± 0.58^{b}	356.33 ± 1.53^{i}	424.33 ± 8.02^{bc}
	T. dicoccum	0.686 ± 0.003^{a}	0.009 ± 0.001^{a}	0.072 ± 0.003^{a}	0.084 ± 0.002^{b}	0.247 ± 0.004^{a}	117.00 ± 1.00^{b}	341.00 ± 4.00^{i}	427.67 ± 8.33^{bc}
	T. speltoides	0.828 ± 0.007^{cd}	0.015 ± 0.001^{ab}	0.105 ± 0.003^{cd}	0.087 ± 0.001^{b}	0.306 ± 0.002^{c}	127.33 ± 1.53^{b}	393.33 ± 2.52^{j}	384.67 ± 14.64^{a}
	T. boeoticum	0.811 ± 0.009^{cd}	0.014 ± 0.001^{a}	0.087 ± 0.002^{b}	0.082 ± 0.002^{b}	0.290 ± 0.002^{b}	120.67 ± 1.53^{b}	385.67 ± 2.08^{j}	379.33 ± 13.31^{a}

* Different letters in the columns indicate significant differences among wheat types ($p<0.05$).

Table S1.7. Enzymatic and non-enzymatic antioxidant defense system responses in shoot against combined salt treatments

Salt treatments	***Wheats***	***SOD*** (U mL^{-1} protein)	***CAT*** (U mL^{-1} protein)	***GR*** (U mL^{-1} protein)	***GST*** (U mL^{-1} protein)	***APX*** (U mL^{-1} protein)	***PRO*** (nmol gr^{-1} fw)	***MDA*** (nmol gr^{-1} fw)	***Total Protein*** (µg mL^{-1} protein)
Control	*T. monococcum*	0.743 ± 0.035[b]	0.024 ± 0.009[bc]	0.081 ± 0.001[ab]	0.081 ± 0.003[b]	0.294 ± 0.007[bc]	50.83 ± 1.27[a]	17.23 ± 0.29[a]	462.67 ± 15.01[cd]
	T. dicoccum	0.783 ± 0.008[bc]	0.011 ± 0.001[a]	0.077 ± 0.001[ab]	0.078 ± 0.001[ab]	0.272 ± 0.002[ab]	57.33 ± 1.15[a]	16.00 ± 1.00[a]	490.67 ± 13.32[cd]
	T. speltoides	0.884 ± 0.005[de]	0.015 ± 0.001[ab]	0.114 ± 0.002[d]	0.114 ± 0.002[cd]	0.323 ± 0.002[cd]	75.33 ± 1.53[ab]	18.67 ± 0.58[a]	413.67 ± 11.59[b]
	T. boeoticum	0.821 ± 0.008[cd]	0.016 ± 0.001[ab]	0.096 ± 0.002[c]	0.108 ± 0.002[c]	0.310 ± 0.002[c]	71.00 ± 1.00[ab]	17.33 ± 0.58[a]	433.67 ± 8.73[ab]
50 mM NaCl + 50 mM KCl	*T. monococcum*	0.918 ± 0.002[e]	0.010 ± 0.001[a]	0.119 ± 0.002[d]	0.102 ± 0.003[c]	0.338 ± 0.002[d]	562.67 ± 1.15[l]	41.67 ± 0.58[b]	579.33 ± 8.08[gh]
	T. dicoccum	0.877 ± 0.006[de]	0.023 ± 0.001[bc]	0.120 ± 0.006[d]	0.112 ± 0.002[cd]	0.316 ± 0.003[c]	592.33 ± 3.21[lm]	37.00 ± 1.00[b]	575.00 ± 3.61[g]
	T. speltoides	1.129 ± 0.006[j]	0.020 ± 0.001[b]	0.194 ± 0.004[g]	0.130 ± 0.002[de]	0.377 ± 0.002[f]	637.00 ± 6.24[m]	57.00 ± 1.00[c]	556.33 ± 3.78[g]
	T. boeoticum	1.054 ± 0.008[i]	0.018 ± 0.001[b]	0.186 ± 0.002[fg]	0.129 ± 0.002[de]	0.357 ± 0.004[e]	633.33 ± 3.79[m]	55.33 ± 1.53[c]	543.00 ± 4.35[f]
100 mM NaCl + 100 mM KCl	*T. monococcum*	0.737 ± 0.003[b]	0.024 ± 0.002[bc]	0.075 ± 0.003[a]	0.076 ± 0.001[ab]	0.282 ± 0.009[b]	371.67 ± 0.58[gh]	153.67 ± 0.58[ef]	499.67 ± 10.78[e]
	T. dicoccum	0.708 ± 0.007[a]	0.012 ± 0.001[a]	0.074 ± 0.002[a]	0.092 ± 0.002[bc]	0.274 ± 0.004[b]	465.67 ± 2.08[j]	132.67 ± 3.06[e]	506.00 ± 13.45[e]
	T. speltoides	0.923 ± 0.014[e]	0.015 ± 0.001[ab]	0.108 ± 0.004[cd]	0.097 ± 0.002[bc]	0.335 ± 0.003[d]	494.00 ± 2.65[jk]	175.00 ± 3.61[f]	494.33 ± 9.07[e]
	T. boeoticum	0.883 ± 0.006[de]	0.015 ± 0.001[ab]	0.097 ± 0.003[c]	0.083 ± 0.002[b]	0.325 ± 0.003[d]	475.33 ± 5.86[j]	169.33 ± 3.06[f]	455.33 ± 10.06[c]
150 mM NaCl + 500 uM GB	*T. monococcum*	0.866 ± 0.004[d]	0.013 ± 0.001[a]	0.125 ± 0.001[de]	0.115 ± 0.002[d]	0.321 ± 0.005[cd]	370.00 ± 4.58[gh]	253.33 ± 2.08[g]	564.00 ± 3.61[g]
	T. dicoccum	0.807 ± 0.006[c]	0.024 ± 0.001[bc]	0.124 ± 0.002[de]	0.126 ± 0.002[de]	0.301 ± 0.004[c]	405.00 ± 3.00[hi]	229.67 ± 1.53[g]	581.33 ± 7.51[gh]
	T. speltoides	0.950 ± 0.004[f]	0.034 ± 0.002[d]	0.189 ± 0.003[g]	0.142 ± 0.004[e]	0.346 ± 0.003[de]	456.33 ± 1.53[j]	263.33 ± 2.08[gh]	534.33 ± 5.85[f]
	T. boeoticum	0.931 ± 0.005[ef]	0.032 ± 0.001[cd]	0.174 ± 0.002[fg]	0.129 ± 0.003[de]	0.336 ± 0.004[d]	440.00 ± 3.61[i]	254.00 ± 1.00[g]	535.67 ± 3.51[f]
150 mM KCl + 500 uM GB	*T. monococcum*	0.875 ± 0.003[de]	0.013 ± 0.001[a]	0.127 ± 0.003[de]	0.109 ± 0.004[c]	0.312 ± 0.005[c]	407.67 ± 3.79[hi]	257.33 ± 2.08[g]	590.67 ± 3.05[h]
	T. dicoccum	0.809 ± 0.006[c]	0.018 ± 0.001[b]	0.118 ± 0.004[d]	0.150 ± 0.004[f]	0.305 ± 0.004[c]	436.33 ± 2.52[i]	238.33 ± 3.21[g]	604.67 ± 4.04[h]
	T. speltoides	1.056 ± 0.003[i]	0.030 ± 0.001[c]	0.165 ± 0.003[f]	0.169 ± 0.003[g]	0.351 ± 0.004[e]	468.00 ± 3.61[j]	264.33 ± 4.16[gh]	595.67 ± 2.08[h]
	T. boeoticum	0.998 ± 0.004[g]	0.032 ± 0.001[cd]	0.163 ± 0.003[f]	0.115 ± 0.004[d]	0.310 ± 0.008[c]	439.33 ± 4.16[i]	265.33 ± 0.58[gh]	593.67 ± 7.37[h]
200 mM NaCl + 500 uM GB	*T. monococcum*	0.878 ± 0.004[de]	0.021 ± 0.001[b]	0.093 ± 0.002[bc]	0.096 ± 0.002[bc]	0.303 ± 0.006[c]	208.33 ± 2.52[d]	314.67 ± 1.53[i]	543.33 ± 3.21[f]
	T. dicoccum	0.807 ± 0.006[c]	0.016 ± 0.001[ab]	0.079 ± 0.001[ab]	0.110 ± 0.002[c]	0.259 ± 0.005[a]	242.67 ± 2.08[e]	303.67 ± 1.53[hi]	558.67 ± 3.05[g]
	T. speltoides	0.933 ± 0.005[ef]	0.031 ± 0.001[c]	0.145 ± 0.003[ef]	0.120 ± 0.002[d]	0.305 ± 0.005[c]	274.33 ± 3.51[ef]	347.33 ± 2.08[i]	530.33 ± 4.16[f]
	T. boeoticum	0.929 ± 0.003[ef]	0.032 ± 0.001[cd]	0.134 ± 0.002[e]	0.111 ± 0.005[cd]	0.297 ± 0.003[bc]	264.67 ± 1.53[e]	336.00 ± 2.00[i]	520.67 ± 3.51[ef]
200 mM KCl + 500 uM GB	*T. monococcum*	0.790 ± 0.003[c]	0.017 ± 0.001[ab]	0.087 ± 0.002[b]	0.113 ± 0.003[cd]	0.284 ± 0.004[b]	202.33 ± 1.53[d]	276.33 ± 2.52[h]	599.00 ± 5.01[h]
	T. dicoccum	0.731 ± 0.003[b]	0.014 ± 0.001[a]	0.082 ± 0.002[ab]	0.135 ± 0.003[e]	0.274 ± 0.003[b]	209.33 ± 3.51[d]	270.00 ± 2.65[h]	618.33 ± 4.51[i]
	T. speltoides	0.894 ± 0.002[e]	0.023 ± 0.001[bc]	0.141 ± 0.003[e]	0.145 ± 0.003[e]	0.315 ± 0.004[c]	221.33 ± 5.03[d]	300.00 ± 3.61[h]	578.33 ± 8.08[gh]
	T. boeoticum	0.874 ± 0.003[de]	0.021 ± 0.001[b]	0.109 ± 0.004[cd]	0.107 ± 0.003[c]	0.313 ± 0.003[c]	208.33 ± 4.16[d]	293.67 ± 3.79[h]	638.00 ± 4.00[ij]
50 mM NaCl + 50 mM KCl + 500 uM GB	*T. monococcum*	0.993 ± 0.003[g]	0.017 ± 0.001[ab]	0.155 ± 0.002[f]	0.120 ± 0.003[d]	0.395 ± 0.004[g]	679.00 ± 8.54[n]	27.33 ± 1.15[ab]	629.00 ± 3.61[i]
	T. dicoccum	0.939 ± 0.004[ef]	0.032 ± 0.001[cd]	0.163 ± 0.003[f]	0.144 ± 0.003[e]	0.386 ± 0.003[fg]	724.33 ± 8.02[o]	25.67 ± 0.58[ab]	689.00 ± 6.24[k]
	T. speltoides	1.185 ± 0.005[k]	0.028 ± 0.001[c]	0.273 ± 0.007[i]	0.168 ± 0.004[g]	0.445 ± 0.004[h]	785.67 ± 9.07[p]	30.00 ± 0.05[ab]	669.67 ± 3.51[jk]
	T. boeoticum	1.117 ± 0.006[j]	0.027 ± 0.001[c]	0.261 ± 0.004[i]	0.163 ± 0.002[g]	0.437 ± 0.007[h]	778.67 ± 10.79[p]	28.33 ± 0.58[ab]	657.67 ± 3.51[j]
100 mM NaCl + 100 mM KCl + 500 uM GB	*T. monococcum*	0.809 ± 0.005[c]	0.030 ± 0.001[c]	0.093 ± 0.002[bc]	0.117 ± 0.005[d]	0.335 ± 0.003[d]	505.33 ± 3.51[k]	101.67 ± 0.58[d]	594.00 ± 6.56[h]
	T. dicoccum	0.787 ± 0.005[c]	0.020 ± 0.001[b]	0.092 ± 0.003[bc]	0.135 ± 0.004[e]	0.309 ± 0.005[c]	570.33 ± 6.66[l]	85.00 ± 1.00[d]	624.00 ± 3.01[i]
	T. speltoides	0.988 ± 0.004[g]	0.026 ± 0.001[c]	0.135 ± 0.004[e]	0.153 ± 0.002[f]	0.364 ± 0.004[e]	604.33 ± 8.74[lm]	103.67 ± 3.79[d]	594.67 ± 2.08[h]
	T. boeoticum	0.939 ± 0.005[ef]	0.027 ± 0.001[c]	0.143 ± 0.003[e]	0.145 ± 0.003[e]	0.347 ± 0.002[de]	597.00 ± 6.00[lm]	99.00 ± 3.61[d]	605.67 ± 2.30[hi]
TOTAL	*T. monococcum*	0.819 ± 0.068[cd]	0.019 ± 0.002[b]	0.098 ± 0.021[c]	0.095 ± 0.017[bc]	0.311 ± 0.030[c]	316.36 ± 159.95[f]	162.52 ± 127.64[f]	534.79 ± 52.87[f]
	T. dicoccum	0.793 ± 0.057[c]	0.017 ± 0.006[ab]	0.096 ± 0.023[c]	0.113 ± 0.024[cd]	0.293 ± 0.031[bc]	350.11 ± 169.48[g]	157.84 ± 122.71[ef]	542.05 ± 61.51[f]
	T. speltoides	0.955 ± 0.097[f]	0.029 ± 0.040[c]	0.158 ± 0.041[f]	0.126 ± 0.027[de]	0.343 ± 0.033[de]	380.16 ± 181.69[h]	189.40 ± 138.37[f]	505.39 ± 70.94[e]
	T. boeoticum	0.903 ± 0.132[e]	0.025 ± 0.003[bc]	0.153 ± 0.064[f]	0.116 ± 0.023[d]	0.329 ± 0.034[d]	368.88 ± 181.64[gh]	182.88 ± 135.59[f]	507.84 ± 72.65[e]

* Different letters in the columns indicate significant differences among wheat types ($p<0.05$).

Table S1.8. Percentage changes in antioxidant enzyme activities in hulled wheats as a result of NaCl applications compared to control

NaCl treatments	***Wheats***	***SOD***	***CAT***	***GR***	***GST***	***APX***	***Proline***	***MDA***	***Carotene***
30 mM NaCl	*T. monococcum*	18	206	61	64	28	687	186	18
	T. dicoccum	24	216	81	88	35	771	211	48
	T. speltoides	20	295	104	8	22	498	274	21
	T. boeoticum	29	260	169	9	30	626	257	19
50 mM NaCl	*T. monococcum*	27	272	116	95	43	766	310	43
	T. dicoccum	10	293	144	200	54	827	318	59
	T. speltoides	-1	348	208	66	41	757	395	68
	T. boeoticum	3	308	261	52	49	783	399	79
100 mM NaCl	*T. monococcum*	21	187	105	62	24	1275	1496	31
	T. dicoccum	-1	231	102	158	35	1082	1530	50
	T. speltoides	1	285	230	33	19	899	1755	76
	T. boeoticum	12	263	347	30	24	971	1758	90
150 mM NaCl	*T. monococcum*	15	92	65	28	4	669	2749	31
	T. dicoccum	-6	121	69	68	12	686	2869	53
	T. speltoides	-9	208	150	116	2	538	2853	67
	T. boeoticum	1	180	189	-11	15	593	2948	75
200 mM NaCl	*T. monococcum*	-3	-14	-10	-13	-11	233	3939	26
	T. dicoccum	-14	4	-7	17	-6	237	4233	35
	T. speltoides	-19	144	24	-56	-14	179	3928	70
	T. boeoticum	-8	117	49	-56	-8	200	4090	76

Table S1.9. Percentage changes in antioxidant enzyme activities in hulled wheats as a result of KCl applications compared to control

KCl treatments	***Wheats***	***SOD***	***CAT***	***GR***	***GST***	***APX***	***Proline***	***MDA***	***Carotene***
30 mM KCl	*T. monococcum*	9	35	34	20	10	640	191	17
	T. dicoccum	-5	33	51	63	23	622	217	48
	T. speltoides	-23	187	78	-25	11	511	197	15
	T. boeoticum	-12	157	136	-23	19	556	193	24
50 mM KCl	*T. monococcum*	14	97	92	47	20	1137	319	35
	T. dicoccum	1	105	93	113	34	1113	321	67
	T. speltoides	9	278	158	1	24	909	429	55
	T. boeoticum	-10	245	245	7	40	969	394	74
100 mM KCl	*T. monococcum*	17	43	78	29	15	1505	1431	48
	T. dicoccum	-1	26	82	95	23	1430	1538	85
	T. speltoides	20	230	126	-14	17	1138	1639	90
	T. boeoticum	33	198	179	-8	22	1244	1680	111
150 mM KCl	*T. monococcum*	13	-4	61	7	10	1100	3153	45
	T. dicoccum	-7	-18	65	80	18	1048	3298	76
	T. speltoides	10	158	61	-29	5	810	3430	86
	T. boeoticum	14	264	128	-30	17	866	3550	103
200 mM KCl	*T. monococcum*	-7	-13	-4	-8	-17	241	3753	42
	T. dicoccum	-24	-20	0	54	-10	236	4130	76
	T. speltoides	-18	108	19	-47	-7	149	3831	80
	T. boeoticum	-11	92	31	-51	0	159	4008	98

Table S1.10. Percentage changes in antioxidant enzyme activities compared to control as a result of co-application of NaCl and KCl and supplementation with exogenous GB

Combined Salt and GB treatments	*Wheats*	*SOD*	*CAT*	*GR*	*GST*	*APX*	*Proline*	*MDA*	*Carotene*
NaCl50+KCl50	*T. monococcum*	47	206	140	91	35	1921	315	57
	T. dicoccum	23	218	158	141	41	1825	316	101
	T. speltoides	48	193	195	28	24	1413	398	93
	T. boeoticum	58	150	310	31	33	1553	395	128
NaCl100+KCl100	*T. monococcum*	1	24	4	-2	1	1484	1535	77
	T. dicoccum	-22	20	20	49	15	1550	1514	114
	T. speltoides	-7	130	54	-41	-2	1168	1653	114
	T. boeoticum	-2	102	116	-49	18	1268	1682	130
NaCl50+KCl50+GB500	*T. monococcum*	63	331	238	156	80	2315	167	110
	T. dicoccum	39	357	286	251	103	2293	200	164
	T. speltoides	61	304	302	76	76	1783	158	158
	T. boeoticum	75	271	457	90	95	1956	164	192
NaCl100+KCl100+GB500	*T. monococcum*	18	173	36	99	47	2045	897	101
	T. dicoccum	-3	187	55	161	57	2069	916	153
	T. speltoides	-22	272	112	34	35	1552	871	179
	T. boeoticum	17	269	194	25	50	1728	907	204

Table S1.11. Percentage changes in antioxidant enzyme activities of NaCl and KCl applications supplemented with exogenous GB compared to the control

Salt with GB supplement	***Wheats***	***SOD***	***CAT***	***GR***	***GST***	***APX***	***Proline***	***MDA***	***Carotene***
NaCl150+GB500	*T. monococcum*	30	170	118	83	42	1192	2564	49
	T. dicoccum	0	225	127	116	40	1156	2624	67
	T. speltoides	1	330	190	10	18	927	2536	110
	T. boeoticum	16	275	270	11	27	1005	2612	126
NaCl200+GB500	*T. monococcum*	21	43	42	51	30	625	3240	54
	T. dicoccum	-2	87	27	83	15	630	3543	69
	T. speltoides	-8	119	57	-16	-3	508	3206	125
	T. boeoticum	3	531	107	-12	5	568	3324	142
KCl150+GB500	*T. monococcum*	29	72	158	88	51	1350	2446	71
	T. dicoccum	3	86	153	194	71	1293	2604	113
	T. speltoides	19	308	139	59	66	1004	2442	145
	T. boeoticum	29	275	214	16	57	1084	2526	171
KCl200+GB500	*T. monococcum*	12	111	37	83	33	613	2840	80
	T. dicoccum	-9	91	33	147	48	562	3153	109
	T. speltoides	-4	217	76	22	22	418	2956	137
	T. boeoticum	6	209	118	-13	42	409	3052	161
